# Impact of Climate and Slope Aspects on the Composition of Soil Bacterial Communities Involved in Pedogenetic Processes along the Chilean Coastal Cordillera

**DOI:** 10.3390/microorganisms10050847

**Published:** 2022-04-20

**Authors:** Victoria Rodriguez, Lisa-Marie Moskwa, Rómulo Oses, Peter Kühn, Nicolás Riveras-Muñoz, Oscar Seguel, Thomas Scholten, Dirk Wagner

**Affiliations:** 1 GFZ German Research Centre for Geosciences, Section Geomicrobiology, 14473 Potsdam, Germany; vrodrigu@gfz-potsdam.de (V.R.); lmoskwa@gmx.de (L.-M.M.); 2 Centro Regional de Investigación y Desarrollo Sustentable de Atacama, Universidad de Atacama (CRIDESAT UDA), Copayapu 484, Copiapó 1530000, Chile; romulo.oses@uda.cl; 3 Department of Geosciences, Soil Science and Geomorphology, University of Tübingen, 72070 Tübingen, Germany; peter.kuehn@uni-tuebingen.de (P.K.); nicolas-andres.riveras-munoz@uni-tuebingen.de (N.R.-M.); thomas.scholten@uni-tuebingen.de (T.S.); 4 Facultad de Ciencias Agronómicas, Universidad de Chile, Av. Santa Rosa #11315, La Pintana, Santiago 8820808, Chile; oseguel@uchile.cl; 5 Institute of Geosciences, University of Potsdam, 14476 Potsdam, Germany

**Keywords:** bacterial-community structure, bacterial diversity, climate gradient, slope aspect, Chilean Coastal Cordillera, soil formation

## Abstract

Soil bacteria play a fundamental role in pedogenesis. However, knowledge about both the impact of climate and slope aspects on microbial communities and the consequences of these items in pedogenesis is lacking. Therefore, soil-bacterial communities from four sites and two different aspects along the climate gradient of the Chilean Coastal Cordillera were investigated. Using a combination of microbiological and physicochemical methods, soils that developed in arid, semi-arid, mediterranean, and humid climates were analyzed. *Proteobacteria*, *Acidobacteria*, *Chloroflexi*, *Verrucomicrobia*, and *Planctomycetes* were found to increase in abundance from arid to humid climates, while *Actinobacteria* and *Gemmatimonadetes* decreased along the transect. Bacterial-community structure varied with climate and aspect and was influenced by pH, bulk density, plant-available phosphorus, clay, and total organic-matter content. Higher bacterial specialization was found in arid and humid climates and on the south-facing slope and was likely promoted by stable microclimatic conditions. The presence of specialists was associated with ecosystem-functional traits, which shifted from pioneers that accumulated organic matter in arid climates to organic decomposers in humid climates. These findings provide new perspectives on how climate and slope aspects influence the composition and functional capabilities of bacteria, with most of these capabilities being involved in pedogenetic processes.

## 1. Introduction

Soils play a relevant role both in the functioning of the environment and in life sustainability [1,2]. Soil is a mixture of minerals and organic matter and can be understood as a product of the interaction between biota, climate, topography, and parent materials, the balance of which can change over time [3]. Within biota, microbial communities play a fundamental role in soil formation—also called pedogenesis—since these communities are responsible for most biological-transformation processes, including mineral weathering, aggregate formation and stabilization, organic-matter accumulation, decomposition, and the succession of terrestrial biodiversity [4,5,6,7,8]. In recent years, there has been remarkable interest in studying the role of microbial communities both in pedogenetic processes along soil transects and in chronosequences of extreme environments, such as deserts, ice-free oases in Antarctica, glacier forefields, permafrost, and volcanic deposits [9,10,11,12,13,14,15]. Findings reveal that progressive pedogenesis and ecosystem development are closely associated with microbial-community change [16,17]. However, despite the increasing knowledge acquired in this field, there is a lack of understanding about how the structure of microbial communities responds to changing climatic conditions or topography and also about how these changes impact pedogenetic processes and ecosystem development or resilience [18,19].

Climate is one of the dominant factors that control soil formation and stabilization [20]. Climatic factors—such as temperature and precipitation—affect edaphic soil properties by influencing organic-matter content, clay, runoff coefficients, infiltration, erosion, and soil-structure formation [18,21,22]. These climate factors shape natural gradients, thereby promoting both habitat diversification and filtering at different spatial scales [8,23]. Previous studies of soil properties along climate gradients have found that increased soil-water availability lowers both soil pH and bulk density while increasing both soil-organic-matter accumulation and the abundance and diversity of bacterial communities [6,18,19,20,24]. These findings indicate that environmental filtering along climate gradients is a determining factor in microbial-community distribution and pedogenesis.

In addition to regional climate, aspect is a crucial topographic factor that affects microclimatic conditions through the amount of solar radiation and precipitation received, thereby regulating temperature, evapotranspiration capacity, and water availability in the local soil [25,26]. These factors influence pedogenetic and erosive processes, thereby affecting nutrient availability, soil pH, plant growth, soil-organic-matter decomposition, and soil fertility and therefore also the stability of the soil ecosystem [25,27,28,29]. For example, north-facing slopes are subject to higher solar radiation than are south-facing slopes of the Southern Hemisphere, thereby resulting in higher soil-surface temperature, less water retention, and less plant coverage [30]. These factors in the north-facing slope promote slower weathering and soil-forming processes, thinner soil horizons, and generally less soil-organic-matter content [19,30]. In general, changes in plant communities and soil-chemical properties caused by aspects can impact the abundance, structure, and functions of bacterial communities on a local scale [18,28,31].

The Chilean Coastal Cordillera provides a large latitudinal extension that covers a wide climate gradient ranging—from north to south—from extremely arid to semi-arid, mediterranean, and cold and humid climates. This gradient is characterized by tremendous diversity of ecological niches and a high level of environmental heterogeneity on the same bedrock [19]. These characteristics make the Chilean Coastal Cordillera one of the best natural laboratories for studying the impact of microbial communities on pedogenesis and the relation of these communities both to differences in the regional climate (gradient) and to differences in the local climatic conditions (aspect). Along this transect, research focusing on the impact of climate on microbial communities has mainly been conducted in the Atacama Desert, where an abundant oligotrophic microbial community has been identified despite hyper-arid conditions [32,33,34,35,36]. While a large amount of information on microbial communities in the Atacama Desert is available, studies have mainly focused on the climatic adaptation of microorganisms and ignored the pedogenetic role that these organisms play.

In considering the crucial role of pedogenetic processes in soil formation and ecosystem development, the following question arose: What is the impact of regional climate changes (gradient) and local climate changes (aspect) on bacterial communities that are involved in soil formation and stabilization? With this question as a starting point, the main objective of the present study was to determine changes in the diversity, structure, and degree of specialization of bacterial communities on the north- and south-facing slopes along a climatic gradient on the Chilean Coastal Cordillera in order to better understand the role that these communities play in pedogenetic processes. For this purpose, samples from four different study areas along the Coastal Cordillera—that is, Pan de Azúcar National Park, Santa Gracia Natural Reserve, La Campana National Park, and Nahuelbuta National Park—were used for 16S rRNA high-throughput sequencing, soil physical analyses, and soil chemical analyses. The results highlight the diversity and functional capabilities of soil bacteria, which shifted from pioneers that accumulated organic matter in arid climates to organic decomposers in humid climates.

## 2. Materials and Methods

### 2.1. Study Sites and Soil Sampling

**Figure 1:**
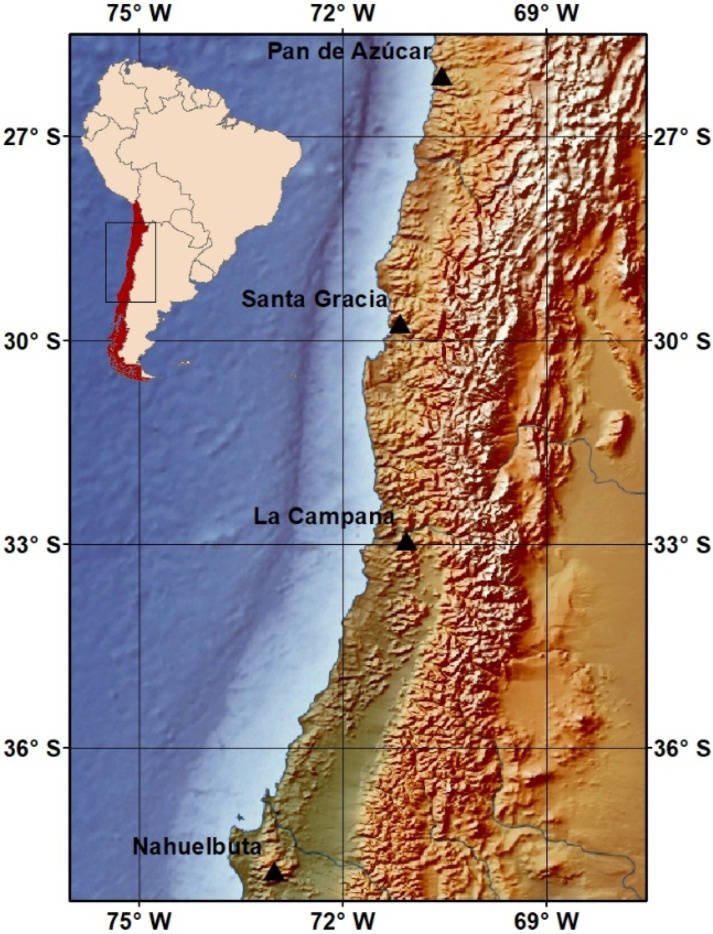
Four study sites located in the Chilean Coastal Cordillera comprising arid soils of Pan de Azúcar, semi-arid soils of Santa Gracia, mediterranean soils of La Campana, and humid-temperate soils of Nahuelbuta.

The research was carried out at four primary study areas (PSAs) along the Chilean Coastal Cordillera (Figure 1) within the framework of the EarthShape priority program (German Science Foundation SPP 1803). According to Muñoz et al. [37], PSAs represent four climatic regions, which correspond to an arid climate in Pan de Azúcar National Park (AZ; −26.1102 S, −70.5493 W), a semi-arid climate in Santa Gracia Natural Reserve (SG; −29.7574 S, −75.1663 W), a mediterranean climate in La Campana National Park (LC; −32.9559 S, −71.0635 W), and a humid climate in Nahuelbuta National Park (NA; −37.8077 S and −73.0135). The mean annual temperature, mean annual precipitation, and altitude of each site are 16.8 °C, 12 mm, and 329–351 m.a.s.l. in AZ, 13.7 °C, 66 mm, and 642–720 m.a.s.l. in SG, 14.1 °C, 367 mm, and 708–732 m.a.s.l. in LC, and 6.6 °C, 1469 mm, and 1200–1270 m.a.s.l. in NA, respectively [19]. Detailed meteorological data and a soil classification and characterization of each PSA were published by Bernhard et al. [19] and Übernickel et al. [38].

Soil samples were collected from the northern and southern mid-slope positions (the NFS and SFS, respectively) along the Chilean Coastal Cordillera during spring 2016. Sixteen samples per PSA were collected, which represented two slope aspects and four different depth increments with two biological replicates each (with a distance of 50–100 m). The soil-depth increments were 0–5 cm, 5–10 cm, 10–20 cm, and 20–40 cm. The sampling method allowed the analysis of regional variations between PSAs along the climate gradient, as well as the influence of slope aspects on the microbial communities. The soil samples were taken devoid of vegetation in order to reduce the impact of the dominant vegetation on the abundance and diversity of the microbial communities. Samples were sieved at 2 mm and stored at −20 °C for further analysis.

### 2.2. Soil Chemical Analysis

A methodology for and results from both soil physical analyses and soil chemical analyses were detailed by Bernhard et al. [19]. From the parameters previously addressed in these authors’ publication, ten parameters—corresponding to soil texture (sand, silt, and clay), pH (determined with a 0.01 M CaCl_2_ solution), plant-available phosphorus (plant-available P), bulk density (BD), the carbon:nitrogen ratio (C/N), total nitrogen (N_t_), total sulfur (S_t_), and pedogenic oxides in the soil (Fe_ox_/Fe_d_)—had a significant effect on the bacterial community and were used for further analysis in the present investigation [19] (Supplemental Material Table S1). In addition, the total organic carbon (TOC) of all samples was determined by Potsdamer Wasser- und Umweltlabor GmbH (PWU, Potsdam, Germany) according to DINEN ISO/IEC 17025:2005.

### 2.3. DNA Extraction and Sequencing

Total genomic DNA was extracted using the PowerMax DNA isolation kit (sixteen samples for AZ) and the PowerSoil DNA isolation kit (sixteen samples per each PSA; SG, LC, and NA) (both from Qiagen, Hilden, Germany) according to the manufacturer’s protocol. Each sample consists of three pooled DNA extraction technical replicates and a PCR in duplicate prior to sequencing. The V4 region of the bacterial small-subunit rRNA gene (16S) was PCR-amplified using barcode-tagged multiplex identifiers—described by Caporaso et al. [39]—that corresponded to 515-F (GTG CCA GCM GCC GCG GTA A) and 806-R (GGA CTA CGV GGG TWT CTA AT). PCR amplification was performed in 25 µL reactions containing 0.75 µL of each primer (20 µM), 0.25 µL of Optitaq DNA polymerase (Roboklon, Berlin, Germany), 2.5 µL of 10× Pol Buffer (C), 1.5 µL of MgCl_2_ (25 mM), 0.25 µL of BSA (20 mg/mL), and 1 µL of dNTP mix (5 mM). The amplification process was performed in a T100^TM^ Thermal Cycler (Bio-Rad Laboratories Inc., Hercules, CA, USA) with the following cycling program: initial denaturation at 95 °C for 5 min followed by 10 cycles of denaturation at 95 °C for 30 s, annealing for 45 s with decreasing temperatures from 65 to 55 °C (decreasing 1 °C/cycle), elongation at 72 °C for 1 min, 30 cycles of denaturation at 95 °C for 1 min, annealing at 55 °C for 30 s, elongation at 72 °C for 1 min, and finally, an elongation step of 7 min at 72 °C. PCR products were purified using carboxyl-coated magnetic beads (Agencourt^®^ AMPure^®^ XP Kit, Beckman Coulter, Brea, CA, USA) following the manufacturer’s recommendations. All amplification products were pooled equitably in a final concentration of 30 ng of DNA and sequenced together. Sequencing of the pooled samples was performed using HiSeq high-throughput sequencing of bacterial and archaeal 16S rRNA genes and was conducted on an Illumina HiSeq (2 × 250 bp) by Eurofins GATC Biotech (Eurofins Scientific, Constance, Germany).

### 2.4. Data Analysis

Raw data quality was checked using FastQC [40]. Dual indexed reads were demultiplexed using CutAdapt [41]. Sequence filtering, error check, chimera removal, and amplicon-sequence-variant (ASV) identification were executed with dada2 [42]. ASV taxonomy was assigned by referring to the SILVA taxonomy database (v138) using VSEARCH in the QIIME2 platform [43,44,45]. Non-target sequences—including chloroplasts and mitochondria—were removed from the analysis. Raw sequences were submitted to the European Nucleotide Archive (http://www.ebi.ac.uk/ena) with BioProject ID PRJEB38745.

Statistical analyses were performed using 16 samples per site that corresponded to two aspects, two biological replicates, and four depths. All samples were subsampled at 32,000 reads and transformed into relative abundances in order to standardize the data. Biological duplicates were analyzed separately. The physicochemical data were standardized by subtracting the mean and dividing by the standard deviation.

Diversity- and community-structure analyses were performed using R studio version 3.6.1. The defined ASVs were used to calculate alpha-diversity indices using the vegan package [46], which included richness, the Shannon index, and Pielou’s evenness. Kruskal–Wallis was used to test for differences in diversity indices among slopes and PSAs. Pearson correlation analysis was carried out to analyze trends between physicochemical parameters and alpha-diversity indices. The effect of climate and slope aspect on the bacterial structure was analyzed via the PERMANOVA test (*p*-level of significance: <0.05) with 999 permutations using the Adonis function. An indicator-value analysis (or IndVal) was performed that avoided rare taxa (>0.1%) when analyzing habitat specialization. IndVal analysis is based on an ASV’s fidelity, and the relative abundance within a particular PSA is calculated using the “indval” function in the labdsv package [47]. Only ASVs with a significant IndVal value (IndVal > 0.8, *p* < 0.05) were considered as a good indicator of specialization for either PSA. In contrast, ASVs with high incidence (>75% of the samples) and high relative abundance over all sites (>0.1%) were considered arbitrarily as habitat generalists. The taxonomic relative abundances across samples were visualized through bubble plots using the package ggplot2 [48]. Finally, the bacterial-community response to physicochemical parameters was determined via canonical correlation analysis (CCA, *p* < 0.05) using CANOCO 5.0 software (Microcomputer Power, Ithaca, NY, USA) [49].

## 3. Results

### 3.1. Soil Physical Properties and Soil Chemical Properties

The four locations along the transect—that is, the arid climate of AZ, the semi-arid climate of SG, the mediterranean climate of LC, and the humid climate of NA—revealed trends among sampling locations of ten physicochemical parameters [19]. In summary, sand content was higher in SG and LC, followed by AZ, and it was significantly lower in NA for both aspects. Silt content was higher in NA, followed by AZ, LC, and SG, and this content was significantly different between the NFS and the SFS in LC. On the other hand, clay content increased to the south and showed significant differences between slope aspects. Clay content was significantly higher in the NFS of SG and the SFS of NA. The BD was lower in NA for both aspects and was significantly different from all PSAs. Moreover, the BD was significantly higher in the NFS of LC. The TOC increased from north to south in both aspects and was significantly different between all PSAs. In both aspects, pH decreased from alkaline in the north to acidic in the south, where LC and NA showed significant differences between the SFS and the NFS, with higher pH in the NFS for both. S_t_ content decreased, while Fe_ox_/Fe_d_, N_t_, and the C/N ratios increased from north to south. The plant-available P increased from AZ to SG and then decreased to NA. Furthermore, plant-available P and Fe_ox_/Fe_d_ showed a significantly higher SFS value than did the NFS in LC.

### 3.2. Diversity Analysis

High-throughput sequencing resulted in a total of 18.4 million raw reads from all 64 soil samples. After filtering, merging, and removing chimeras, a final number of 14.9 million reads—corresponding to 81.1% of the total—was obtained (Supplemental Material Table S2). Of these reads, 95% were assigned to bacteria, 4.8% were assigned to archaea, and 0.2% were not assigned to any known species. In the NFS, of 5,377,632 total reads, 95.4% were assigned to bacteria, 4.3% were assigned to archaea, and 0.3% were not assigned to any known species. In the SFS, of 9,575,576 total reads, 94.8% were assigned to bacteria, 5.1% were assigned to archaea, and 0.2% were not assigned to any known species. Only bacterial data were included in the subsequent analyses due to the low number of archaeal reads (>2000 reads). The number of reads per sample ranged from 32,785 to 608,846, with a mean value of 221,937 sequences per sample, and these reads were then subsampled at 32,000 sequences. Finally, 39,195 ASVs were calculated, and after taxonomic classification, 1477 putative genera were obtained.

**Figure 2:**
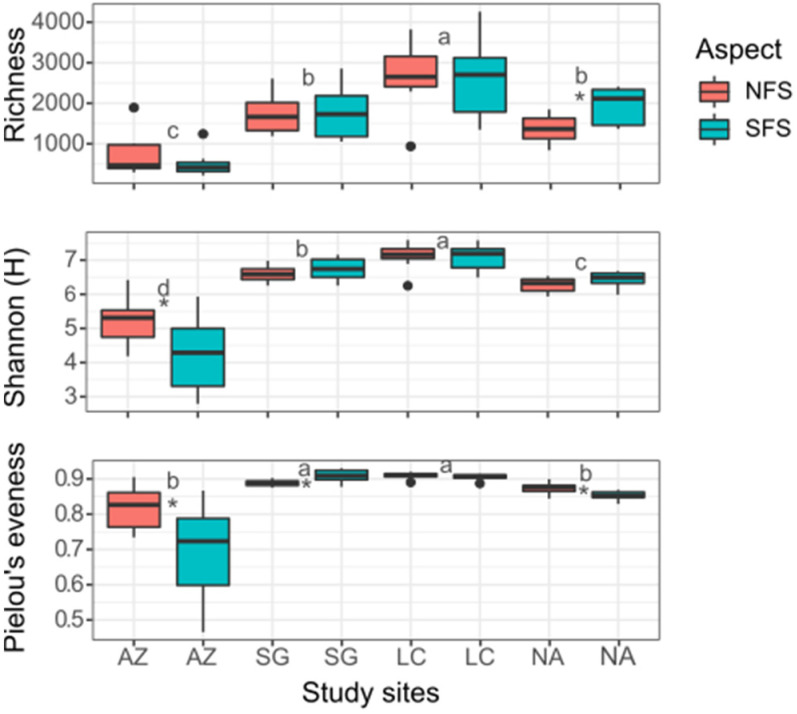
Alpha-diversity measurements of bacterial communities in four sites and two slope aspects. The north and south-facing slopes are represented by NFS and SFS, respectively. Each panel represents one alpha-diversity measure. Richness: total number of ASVs observed; Shannon (H): microbial index of diversity; Pielou’s evenness: equitability. Different letters represent significant differences between sites, while asterisks represent significant differences between aspects within the same site (*p* < 0.05).

Alpha-diversity values were calculated from subsampled ASVs and visualized using R (Figure 2). For both aspects, the richness (number of taxa) was significantly higher in LC (mean: 2633.9) and lower in AZ (mean: 620.4). Regarding depth, richness decreased with depth in all SFS sites, while in the NFS, richness only decreased with depth in AZ and SG. Pielou’s evenness (equitability) showed a mean value of 0.87 for the NFS and 0.83 for the SFS, indicating that the community was equitable and evenly distributed. However, Pielou’s evenness was significantly lower in AZ in both aspects (mean: 0.82 and 0.68 in the NFS and the SFS, respectively), which reveals the higher dominance of some ASVs. The bacterial diversity was high, with an overall Shannon index of mean 6.3. In both aspects, diversity was significantly higher in LC (mean: 7.1), followed by SG (mean: 6.7), NA (mean: 6.4), and AZ (mean: 4.8). Three environmental parameters showed a significant correlation with the Shannon index (Supplemental Material Table S3) in the NFS and corresponded to pH (R = −0.58), C/N (R = 0.47), and St (R = −0.76; *p* < 0.05). For the SFS, pH (R = −0.63), St (R = −0.47), and N_t_ (R = 0.43) were correlated with the Shannon index.

### 3.3. Community-Composition Analysis

**Figure 3:**
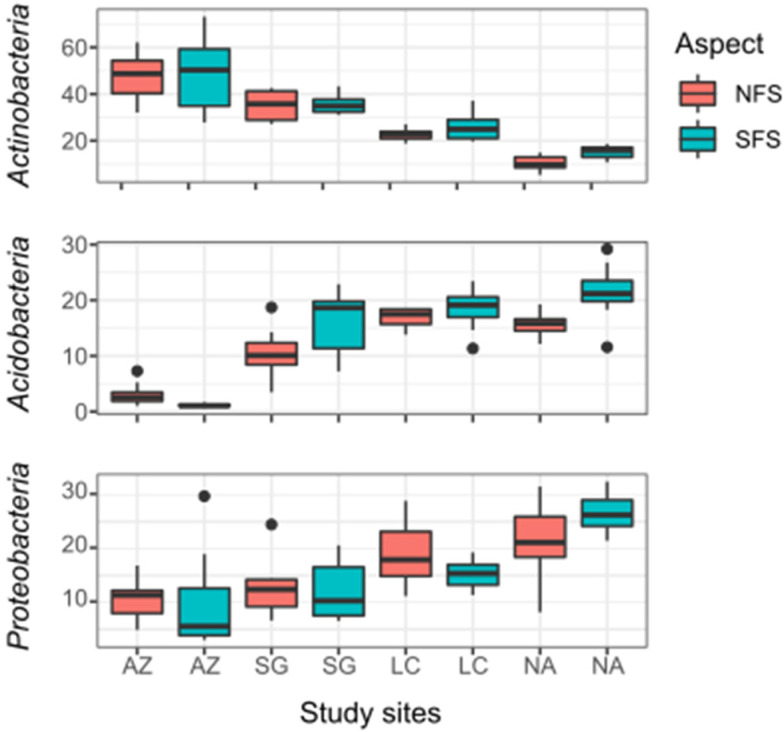
Boxplots of relative abundances of dominant bacteria phyla in four sites and two slope aspects, represented by *Actinobacteria*, *Acidobacteria*, and *Proteobacteria*. The north and south-facing slopes are represented by NFS and SFS, respectively. Each bar represents the sum of all replicates and depth increments of each site/aspect.

Along the climate gradient, seven phyla dominated in both slope aspects: *Actinobacteria* (30.2%), *Proteobacteria* (21%), *Acidobacteria* (12.9%), *Chloroflexi* (9.74%), *Verrucomicrobia* (6.5%), *Planctomycetes* (6.1%), and *Gemmatimonadetes* (4.4%) (Supplemental Material Table S4). These seven phyla represent 86.8% of the total reads in AZ, 90.5% in SG, 91.2% in LC, and 94.5% in NA. In both slope aspects, from north to south, an increase in *Acidobacteria*, *Chloroflexi*, *Planctomycetes*, *Proteobacteria*, and *Verrucomicrobia* and a decrease in *Actinobacteria* and *Gemmatimonadetes* were observed (Figure 3). The most important three phyla in AZ were *Actinobacteria* (48.6%), *Proteobacteria* (15.3%), and *Chloroflexi* (8.8%). In SG and LC, the three dominant phyla were *Actinobacteria* (35.4 and 24.2%, respectively), *Proteobacteria* (17.4 and 22.1%, respectively), and *Acidobacteria* (17.6 and 13.3%, respectively). Finally, NA was dominated by *Proteobacteria* (29.1%), followed by *Acidobacteria* (18.5%) and *Chloroflexi* (16.5%).

PERMANOVA was used to explain differences in bacterial-community structure along the climate gradient and the different slope aspects (Supplemental Material Tables S5 and S6). The four climate sites harbored a community structure that was significantly different (*p* < 0.01), while the NFS and the SFS were significantly different only in the southernmost sites of LC and NA (*p* < 0.05).

**Figure 4:**
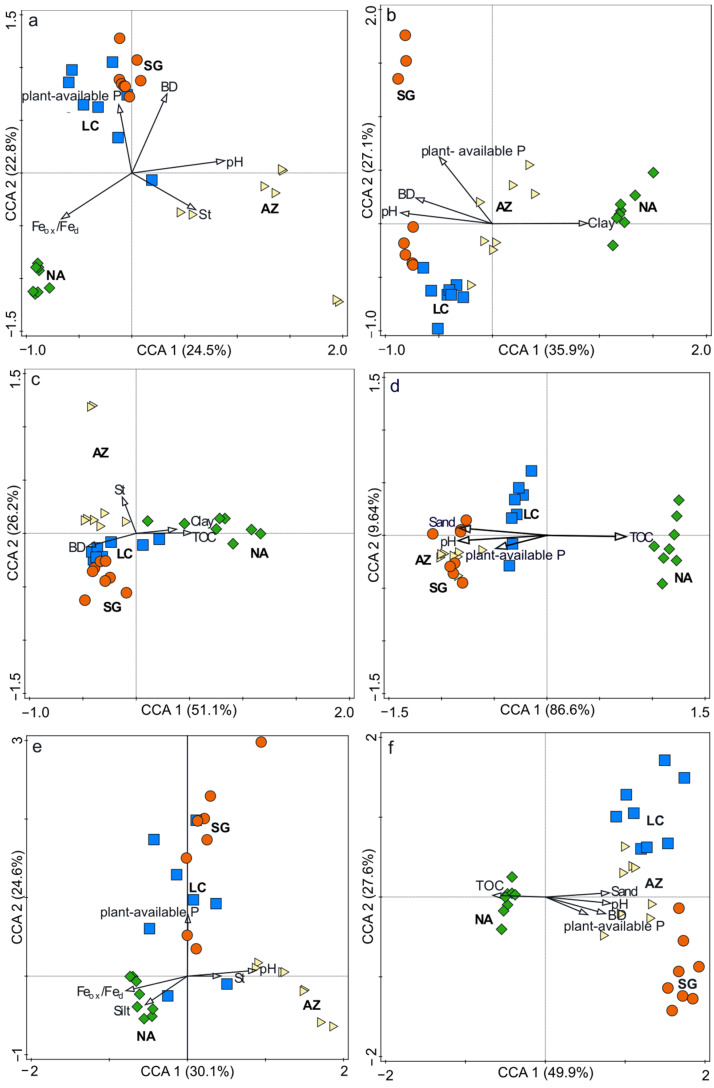
Canonical correspondence analysis (CCA) that correlates environmental parameters with ASV sequencing data in (**a**) the whole community of the NFS, (**b**) the whole community of the SFS, (**c**) habitat generalists in the NFS, (**d**) habitat generalists in the SFS, (**e**) habitat specialists in the NFS, and (**f**) habitat specialists in the SFS. All *p*-values are <0.05. Sites are represented by yellow triangles in AZ, orange circles in SG, blue squares in LC, and green diamonds in NA.

The relation between ASVs and physicochemical parameters was identified using CCA (Figure 4a,b). Results revealed that in the NFS, physiochemical properties explained 29.2% of the overall community-composition variance. Within 29.2%, CCA 1 and 2 explained 24.5% and 22.8% of the total variance, respectively. The graph shows that SG and LC were positively influenced by plant-available P and BD. AZ was influenced by pH, S_t_, and lower levels of weathering, while NA had a positive relation with Fe_ox_/Fe_d_. In the SFS, the explanatory variables accounted for 30% of the total variance of the bacterial distribution, with CCA 1 and 2 explaining 35.9% and 27.1% of this total variance, respectively. The graph reveals that NA was influenced positively by clay content and was influenced negatively by pH and BD.

### 3.4. Bacterial Generalists

**Figure 5:**
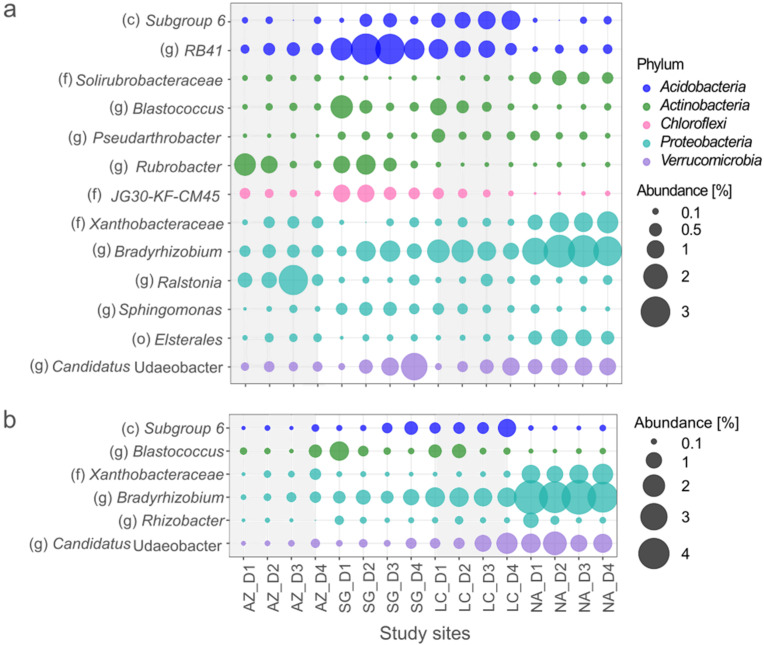
Bubble plots of bacterial generalists at four sites and two slope aspects corresponding to (**a**) the NFS and (**b**) the SFS. For each site, 4 depth profiles—corresponding to D1 (0–5 cm), D2 (5–10 cm), D3 (10–20 cm), and D4 (20–40 cm)—were evaluated. Taxa are shown at the level of (c) class, (o) order, (f) family, and (g) genus.

Of the 39,195 ASVs, only 301 and 302 had a mean of over 0.1% for the NFS and the SFS, respectively. Of these, 16 and 7 were generalists for the NFS and the SFS, respectively, despite the variability of the ecosystems. The generalist community in the NFS was composed of five phyla: *Actinobacteria*, *Proteobacteria*, *Acidobacteria*, *Chloroflexi*, and *Verrucomicrobia*, which together represent thirteen different taxa (Figure 5a). On the other hand, the community in the SFS was composed of four phyla, including *Actinobacteria*, *Proteobacteria*, *Acidobacteria*, and *Verrucomicrobia*, which together represent six different taxa (Figure 5b). The different taxa were found in all sites, but they displayed different site preferences. The *Bradyrhizobium* and *Candidatus* Udaeobacter genera tended to increase from north to south, while the *Rubrobacter* genus decreased. *Acidobacteria* (class *Subgroup 6* and genus *RB41*), the *Blastococcus* genus, and the *JG30-KF-CM45* family displayed a predominant abundance in SG and LC. ASVs from the *Solirubrobacteraceae* family, the *Xanthobacteriaceae order*, the *Rhizobacter* order, and the *Elsterales* class were more abundant in NA. In contrast, the *Ralstonia* genus predominated in AZ. Regarding depth, ASVs from the *Rubrobacter* genus and the *JG30-KF-CM45* family were more abundant on the surface and decreased with depth.

The relation between bacterial generalists and physicochemical parameters explained 55% of the overall community-composition variance in the NFS (Figure 4c). Habitat generalists of SG and LC were influenced mainly by BD, while AZ was influenced by S_t_. In contrast, NA was positively influenced by clay and TOC. Considering the SFS (Figure 4d), physiochemical properties explained 85.3% of the total variance, 86.6% and 9.6% of which were explained by CCA 1 and 2, respectively. NA was separated from all the PSAs. It was positively influenced by TOC and negatively influenced by pH.

### 3.5. Bacteria Specialists

**Figure 6:**
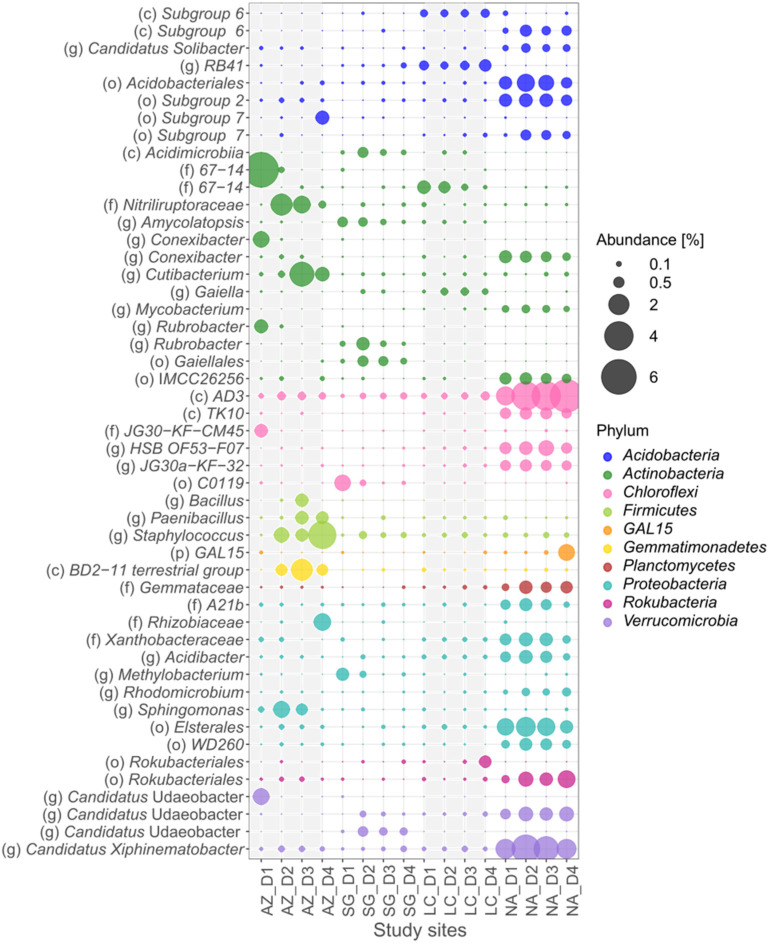
Bubble plot of bacterial specialists at four sites in the NFS. For each site, 4 depth profiles—corresponding to D1 (0–5 cm), D2 (5–10 cm), D3 (10–20 cm), and D4 (20–40 cm)—were evaluated. Taxa are shown at the level of (p) phylum, (c) class, (o) order, (f) family, and (g) genus.

**Figure 7:**
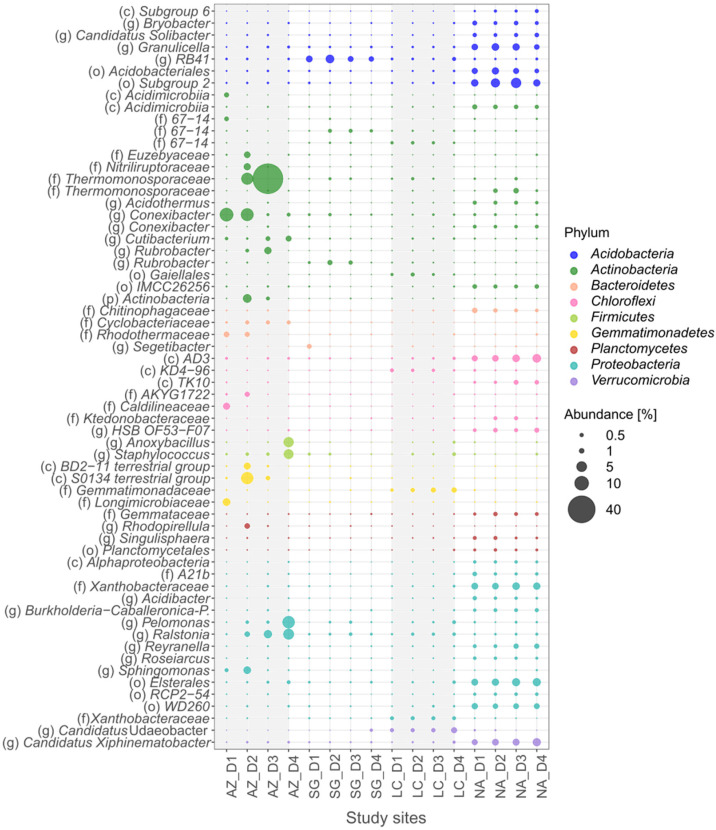
Bubble plot of bacterial specialists at four sites in the SFS. For each site, 4 depth profiles—corresponding to D1 (0–5 cm), D2 (5–10 cm), D3 (10–20 cm), and D4 (20–40 cm)—were evaluated. Taxa are shown at the level of (c) class, (o) order, (f) family, and (g) genus.

Based on the indicator-value analysis (Supplemental Material Table S7), the bacteria specialists in the NFS involved 10 phyla: *Acidobacteria*, *Actinobacteria*, *Chloroflexi*, *Firmicutes*, *GAL 15, Gemmatimonadetes*, *Planctomycetes*, *Proteobacteria*, *Rockubacteria*, and *Verrucomicrobia* (Figure 6). Excluding *GAL 15* and *Rockubacteria*, in addition to the 8 phyla of the NFS, the SFS included *Bacteroidetes* (Figure 7). Of the total specialists, a higher number was in the SFS. Considering the different PSAs, a higher number of specialists was identified in NA (44 in the NFS and 62 in the SFS), followed by AZ (17 in the NFS and 28 in the SFS), LC (5 in the NFS and 8 in the SFS), and finally, SG (7 in the NFS and 5 in the SFS). Of these, the NFS and the SFS shared 18 ASVs in NA (21%), 3 ASVs in AZ (7%), and 0 ASVs in both SG and LC.

*Actinobacteria* was the most dominant and abundant phylum of specialists in both aspects. ASVs from the *Cutibacterium*, *Conexibacter*, and *Rubrobacter* genera and the *67-14* and *Nitriliruptoraceae* families were specialists in AZ in both aspects. Moreover, *Actinobacteria* specialists were found in SG (the *Rubrobacter* genus), LC (the *67-14* family), and NA (the *IMCC26256* order and the *Conexibacter* genus). The family *Thermomonosporaeae* was a prolific specialist in the SFS of AZ, whereas in the NFS, this family was a low-abundant unspecialized ASV. Other specialists were found in both slope aspects, which occupied different PSAs. An example is the *Gaiellales* order, which is a specialist on the NFS of SG and the SFS of LC. The same is true for the *Acidomicrobiia* class, which is a specialist on the NFS of SG and on the SFS of NA.

*Acidobacteria* (ASVs from the *Subgroup 6* class, the *Acidobacteriales* and *Subgroup 2* orders, and the *C. solibacter* genus) were frequently NA specialists in both aspects. Other ASVs from the *Subgroup 6* class and the *RB41* genus were LC specialists. Furthermore, many specialist ASVs were abundant in one of the two aspects. Members of the *Subgroup 7* order were specialists in the NFS of AZ and NA, while the *Bryobacter* and *Granulicella* genera were specialists in the SFS of NA. On the other hand, in *Proteobacteria*, ASVs from the *Elsterales* and *WD260* orders, the *A21b* and *Xanthobacteriaceae* families, and the *Acidibacter* genus were NA specialists, while ASVs from the *Sphingomonas* genus were AZ specialists.

Regarding phyla with a low number of specialists, ASVs from the *AD3* and *TK10* classes and the *HSB OF53-F07* genus that belonged to the *Chloroflexi* phyla were NA specialists, while ASVs from the *C0119* order were SG specialists. *Firmicutes* (ASVs from the *Bacillales* order and the *Staphylococcus* genus) and *Gemmatimonadetes* (from the *BD2-11 terrestrial group* class) were AZ specialists. The family *Gemmataceae* of the *Planctomycetes* phylum were NA specialists. Finally, ASVs from the *Candidatus Xiphinematobacter* genus that belonged to *Verrucomicrobia* phyla were specialists in NA.

The total variance of the NFS specialists was explained by 48.7% of the physicochemical parameters (Figure 4e), 30.1% and 24.6% of which were explained by CCA 1 and CCA 2, respectively. NA was mainly influenced by Fe_ox_/Fe_d_ and silt, while SG and LC were influenced by plant-available P, and AZ was influenced by pH. For the SFS, 61% of the total variance was explained by physicochemical parameters (Figure 4f), 49.9% and 27.6% of which were explained by CCA 1 and 2, respectively. The separation of NA from AZ, SG, and LC was mainly influenced by TOC and was negatively influenced by pH, BD, and sand content.

## 4. Discussion

The impact of both climate and topographic position on soil-formation processes has been recognized and extensively studied both in general [20,24,25,26] and for the Chilean Coastal Cordillera in particular [19]. However, a knowledge gap remains concerning the combined effect of climate gradient and slope aspect on the structure and function of microbial communities, on the one hand, and the effect of these changes on soil-formation processes, on the other hand. Our study provides new evidence on changes in diversity, community structure, and the degree of specialization of microbial communities on the north- and south-facing slopes along the unique climate gradient of the Chilean Coastal Cordillera. Moreover, the study also provides new evidence on the relationship between bacterial communities and pedogenesis.

### 4.1. Climate Gradient

Our findings reveal that the influence of the climate gradient is strongly reflected in the diversity of bacterial communities, which is low in AZ, increases in SG, and reaches a maximum in LC before decreasing again in NA. Our results further indicate that pH and C/N are the most significant parameters in the change in diversity. This result strengthens the findings of previous studies, which have used diverse ecosystem types [7,50,51,52]. Lauber et al. [53] indicated that desert soils with a pH above 8 and temperate-forest soils with a pH < 4.5 have the lowest levels of diversity. An acidic pH is likely the most important challenge for bacterial diversity in NA, while in AZ, the challenge is posed by the combination of a high pH and a low C/N, both of which hamper metabolic processes. A study of cyanobacteria and algae from biological soil crust (BSC) reported a similar diversity pattern when analyzing richness along the gradient [54]. The lower diversity in AZ and NA suggests certain levels of fragility and threatens the stability of ecosystem functionality, especially when facing environmental changes due to the absence of core species that maintain essential functions [55,56].

The microbial-community structure at each site is significantly different from that at the other sites, which highlights the effect of regional climate along the gradient. From arid to humid climates, the microbial-community structure changes according to soil physical properties and soil chemical properties, as already discussed for pH. In addition to the pH, BD, plant-available P, Fe_ox_/Fe_d_, TOC, and clay content play an important role in the development of microbial communities in different climates. These properties are also prominent indicators of progressive soil formation along the Chilean Coastal Cordillera, which suggests an interrelation between community structure and soil-formation processes [19]. For instance, *Actinobacteria* is the most abundant phylum in AZ and decreases toward the humid climate. This phylum dominates in arid soils due to its desiccation resistance and adaptation strategies, as previously reported for the Atacama Desert [32,34,57]. *Actinobacteria* are recognized as pioneers in initial soil environments because they have the ability to metabolize a wide range of substrates as the sole carbon source, thereby playing a pivotal ecological role both in the carbon cycle and in soil formation [9,11,58]. In contrast, *Proteobacteria* and *Acidobacteria* increase from arid to humid climates, reaching their maximum abundance in NA. In general, both phyla increase with increasing TOC (linked to organic C degradation), clay content, microaggregates (<20 µm), recent weathering processes (>Fe_ox_/Fe_d_), and lower plant-available P (efficient P acquisition), which is consistent with the preference of *Actinobacteria* and *Proteobacteria* in NA [16,59,60,61]. These findings suggest that the lifestyle of dominant phyla could contribute to pedogenesis through either biomass generation, weathering, or microaggregate stabilization.

Microorganisms exist as habitat generalists or specialists in response to the climate gradient and to environmental filtering [62]. Generalists show broad environmental tolerance and distribution in many habitats, while specialists have narrow environmental tolerance and a restricted habitat [63,64]. Following this premise, our study identified generalists in the different regional climates, thereby highlighting their high environmental tolerance, better competitiveness, and lower resource requirements [65]. Some *Actinobacteria* with a potential role in nitrogen metabolism—such as *Blastococcus*, *Rubrobacter*, and *Solirubrobacteraceae*—are classified as generalists in different ecosystem types, which include deserts, shrubs, and tree groves [58]. The *Candidatus* Udaeobacter genus of *Verrucomicrobia* belongs to the most abundant soil bacteria worldwide and has a high capacity to produce antibiotics that allow its members to compete efficiently for space and nutrients (e.g., carbon and nitrogen) in soils [66]. Within *Proteobacteria*, *Bradyrhizobium* and *Sphingomonas* have been described as some of the most abundant and ubiquitous genera on six continents due to their multiple survival strategies in soils [17,67]. For instance, *Rhizobiales* (*Bradyrhizobium* and *Xanthobacteriaceae*) can fix nitrogen in symbiotic association with plants or under free-living conditions due to the presence of the *nifH* gene [68,69,70]. The capabilities shown by generalists suggest that generalists contribute to soil-nitrogen availability and that they could improve soil conditions for more demanding species (e.g., vascular plants) or increase aggregate stability [11]. Since generalists are metabolically versatile (i.e., they are adapted to multiple energy resources and carbon sources), they play an important role in maintaining ecosystem functions and stability, especially under changing environmental conditions [64,71].

In addition to the study of generalists, we also examined specialists. AZ harbors a large community of specialists, which are highly adapted bacteria that can survive the extreme environmental conditions of the desert, including the nearly complete lack of organic matter [19,36]. The low concentrations of nitrogen in the parent material promote the colonization and dominance of diazotrophic bacteria, such as *Pelomonas* and *Ralstonia* [14]. *Anoxybacillus*, *Staphylococcus*, and *Paenibacillus* (of the *Bacillus* order) are adapted to the use of recalcitrant carbon substrates and inorganic nutrients, thereby allowing for the accumulation of organic matter [34,68,69]. *Conexibacter* and *67-14* (of the *Solirubrobacterales* order) have been linked to chemosynthetic CO_2_ fixation and ferrous–ferric redox reactions, thereby enhancing weathering and the availability of micronutrients in soils [13,72,73]. The *Sphingomonadacea* family improves soil nutrition by degrading polycyclic aromatic hydrocarbons with hydrophobic properties [74]. *Gemmatimonadetes* that are adapted to low soil moisture have been associated with phosphorous metabolism [12,14,74]. According to these capabilities, bacterial specialists in both slope aspects of AZ are pioneers in terms of organic-matter accumulation during early ecosystem development and thus promote pedogenetic processes [5,10]. This role is complementary to the function of BSC in AZ, which contributes to bio-weathering and to the accumulation of nitrogen and organic carbon, particularly in the upper soil layer [75,76].

Unexpectedly, NA contains the most significant number of specialists linked to acidification and increased TOC, clay, and Fe_ox_/Fe_d_ in the soil. The increased organic matter in this site satisfies the nutrient demand through efficient nutrient recycling via microorganisms [77,78]. Our findings indicate that specialists are involved in decomposition processes. For instance, species of the *Acidobacteria* phylum can hydrolyze polymers such as cellulose, hemicellulose, or starch due to the high content of glycoside hydrolases [79]. From this phylum, *Subgroups 2* and *6*, *Candidatus* Solibacter, *Acidobacteriales*, and *Bryobacter* have been documented in global acidic forest soils [80,81,82]. From *Proteobacteria*, species of the *Burkholderiales* order are efficient mineral-weathering bacteria and are also involved in the decomposition of lignocellulose due to the presence of abundant laccase genes [69,82]. In turn, other *Proteobacteria*, such as *WD260*, *A21b*, and *Acidibacter*, are associated with the decomposition of C-labile compounds, such as glucose and naphthalene [83]. *Alphaproteobacteria* and *Solibacterales* have abundant genes for phosphatase-enzyme synthesis and phosphorous solubilization, which can mobilize phosphorus in soils with a low P content [17]. Finally, we identified specialists that are involved in nitrogen fixation in order to improve soil fertility, including *Burkholderia-Caballeronia*, *Variovorax*, *Xanthobacteraceae*, *Granulicella*, *HSBOF53-F07* (the genus belonging to *Chloroflexi*), *JG30a-KF-32* (the family belonging to *Acidobacteria*), and *Gemmataceae* [16,69,79,81,82,84,85]. The identified potential functions suggest that bacterial specialists in both slope aspects of NA are associated with weathering, with the breakdown of organic compounds and biopolymers as a source of nutrients for plants, and with the improvement of soil stability via the formation of stable aggregates in the topsoil.

AZ and NA are unique hotspots of bacterial specialization along the climate gradient, which suggests that they represent climatically and topographically stable ecosystems [86,87]. Specialized bacteria in AZ could provide evidence of climatic stability and a lack of latitudinal movement since the late Jurassic [88]. On the other end of the transect, NA represents an old, developed, and stable landscape due to climatic stability, prolonged tectonic inactivity, and a lack of large-scale glacial erosion [89]. These attributes have allowed for a vast development of endemic plant species that support bacterial specialization in NA. In contrast, SG and LC harbor a low number of bacterial specialists. Both sites are unstable due to topographic and climatic instability since the Pleistocene and the second half of the Holocene in central Chile (~30–35 S), thereby resulting in high levels of both plant-extinction rates and plant replacement [90,91]. According to these conditions, bacterial specialists could undergo extinction in SG and LC in the face of environmental change, thus resulting in the loss of specific habitat attributes [71].

Overall, our findings indicate that regional climate is a powerful factor in shaping bacterial communities along the Chilean Coastal Cordillera. We suggest that SG and LC are topographically and climatically unstable ecosystems that promote a low number of bacterial specialists and high bacterial diversity in order to cope with environmental disturbances. On the other hand, the ecosystem stability in AZ and NA has allowed the bacterial community there to specialize in the use of specific resources with strong ecological preferences that are linked to the bacteria’s role in both soil formation and ecosystem functioning [87].

### 4.2. Slope Aspect

The slope aspect causes changes in both the diversity and structure of bacterial communities that result from particular microclimatic and physicochemical differences [25,28]. The Shannon index—which indicates bacterial diversity—is only significantly different between AZ in the NFS and the SFS even though neither aspect shows any physicochemical differences. Along the same lines, Bernhard et al. [19] found differences in bacterial abundance between the two aspects in AZ and suggested that these differences were the product of varying radiation intensities. Soil moisture has also been recognized as a critical factor in dry regions [92]. It has been shown, for instance, that small changes in soil moisture have a large impact on microbial diversity in different aspects of the dry valleys of Antarctica [93]. Although AZ is one of the driest places on Earth, small-scale fog mobilized by southwesterly winds during the night provides moisture to the soil, especially in the SFS [94,95]. The differences in soil moisture resulting from the slope aspect could explain the decrease in diversity in the SFS and could also promote the increase in some taxa during wetting [93]. For example, one ASV from the *Thermomonosporaceae* family reaches 49.9% abundance in the SFS compared with 1.4% abundance in the NFS, which indicates a high degree of adaptation to specific microclimatic conditions. In addition to radiation and moisture, the occurrence of BSC cover and the thickness of this cover are important factors that control slope-scale bacterial diversity in arid sites by influencing moisture and physicochemical properties [96,97]. Since BSC coverage in AZ reaches as much as 40% in both aspects, it could impact diversity, as well [19]. Overall, the low bacterial diversity in AZ—especially in the SFS—reinforces our understanding of the fragility of ecosystem functionality, especially under environmental changes that might impact the provision of key ecosystem services, such as soil fertility and biomass production [98].

The bacterial-community structure of the NFS only differs significantly from that of the SFS in the two southernmost sites of LC and NA, which is mediated by aspect-induced changes in soil chemical properties. This finding suggests that the effect of the slope aspect depends on climate and latitude. Latitude accounts for the increasing differences in radiation time between the SFS and the NFS when moving southward and alters aspect-related soil pH, plant-available P, clay content, BD, and Fe_ox_/Fe_d_ [19]. Of these variables, the low soil pH in the SFS is the most critical factor that affects bacterial-community structure, which is likely due to the narrow pH range for optimal bacterial growth [18]. Moreover, soil microorganisms strongly compete with plants for essential nutrients, such as phosphorous, whose lower bioavailability in the NFS could modify the community structure [84]. We suggest that these soil properties in NA have promoted the differentiation of specialists (ASV level) in both slope aspects. However, in contrast to NA, the low number of specialists shared on both slope aspects in AZ may be more related to microclimatic conditions, as was suggested by the observed differences in bacterial diversity. In either case, the specialists that were found in both slope aspects of AZ and NA have the same potential functions described above. The variance of bacterial specialists among aspects reflects these specialists’ high microclimatic specificity and adaptation, yet they still perform the same functions necessary to maintain ecosystem functioning [25,99].

In general, along the gradient, a higher number of generalists was found in the NFS, and a higher number of specialists was found in the SFS. These findings suggest that more stable conditions exist in the SFS than in the NFS. This suggestion is supported by the findings of Pen-Mouratov et al. [31] in the “Evolution Canyon” in Israel. Indeed, these authors demonstrated that the older and more stable climate ecosystem in the NFS of the Northern Hemisphere harbors a higher number of species. In addition, the NFS receives more radiation in the Southern Hemisphere and experiences greater diurnal and seasonal microclimatic changes than does the shaded slope (SFSs), thereby making it more difficult for specialists to survive in the former setting [25]. This decrease in the number of specialists in the NFS may be evidence of a functional biotic-homogenization process in which specialized communities are replaced by generalists with similar or different functions [100]. Although the performance of generalists may be less efficient than that of specialists, a range of species that respond differently to environmental perturbation can stabilize the functioning and processes of the ecosystem on the NFS [99].

Our findings reveal that soil-bacterial diversity and changes to the microbial-community structure depend on local filters that are promoted by the slope aspect. However, the response is not constant along the climate gradient. We suggest that the response of bacterial communities could be considered a result of the interplay between different factors, such as microclimate conditions, soil cover (BSC and/or vegetation), and physicochemical properties, all of which depend primarily on large-scale factors, such as climate and latitude. These findings highlight the importance of the remarkable and unique climate gradient of the Chilean Coastal Cordillera in discriminating among multiple factors that shape microbial communities. Moreover, we encourage similar approaches be taken to predict the function and impact of microbial communities in ecosystem development.

## 5. Conclusions

Our study revealed shifts in the diversity and structure of microbial communities that were induced by climate and aspects along the Chilean Coastal Cordillera, and a correlation between these shifts and prominent indicators of soil formation was found. Along the studied climate gradient, the arid and humid sites behave as stable ecosystems that promote low diversity and high bacterial specialization. Moreover, the south-facing slope represents a more stable ecosystem than the north-facing slope and hosts a greater number of specialists. The presence of bacterial specialists is associated with ecosystem-functional traits. In the arid climate, bacterial specialists are mainly dominated by pioneer microorganisms that are involved in organic-matter accumulation, which is key in the initial pedogenetic process and in ecosystem development. In contrast, the humid-climate bacteria specialists are dominated by microorganisms that are involved in recycling organic compounds as a source of plant nutrients and soil stabilization. Nonetheless, soil-manipulation experiments coupled with transcriptomic analysis would be required in future studies in order to determine whether these bacteria are actively accomplishing this role. In the case of the semi-arid and mediterranean sites, we observed a low number of specialists and a high level of bacterial diversity for coping with the climatic and topographic variability. Our findings provide a basis for future studies on stabilizing, maintaining, and improving different soil ecosystems that are exposed to climate change.

## Data Availability

Raw Illumina sequencing data were submitted to the European Nucleotide Archive (http://www.ebi.ac.uk/ena) with the BioProject ID PRJEB38745 under the accession numbers ERS4643350-ERS4643389 and ERS4652898-ERS4652900 (SequencingRuns: ERR4234140-ERR4234182). The data are not publicly available and will be set public after publication.

## Supplementary Materials

The following supporting information can be downloaded at: https://www.mdpi.com/article/10.3390/microorganisms10050847/s1, Table S1: Overview of selected soil physicochemical data along the depth profiles of the north and south-facing mid-slopes in the four climate regions; Table S2: List of total amplicon sequence variants (ASVs) per sample; Table S3: Pearson correlation of soil bacterial diversity and physicochemical parameters in the north and south-facing mid-slope; Table S4: Relative abundances in % of dominant bacterial phyla obtained in the four climate regions and four depths. Table S5: Results of PERMANOVA analysis using adonis function to compare bacterial community composition between sites and slope aspects; Table S6: Results of PERMANOVA analysis using pairwise adonis test based on Bray–Curtis distances with 999 permutations for comparing bacterial community composition between sites and slope aspects; Table S7: Indicator-value analysis (or IndVal) of bacterial specialists identified in the north- and south-facing mid-slope.
