# Supplementary material for: Impact of Climate and Slope Aspects on the Composition of Soil Bacterial Communities Involved in Pedogenetic Processes along the Chilean Coastal Cordillera
Source: Microorganisms. 2022 Apr 20;10(5):847. doi: 10.3390/microorganisms10050847 (PMC9143490; doi:10.3390/microorganisms10050847)
Supplement: Supplementary file 1 [file microorganisms-10-00847-s001.zip › microorganisms-1653566-supplementary.pdf]

Table S1. Overview of selected soil physicochemical data along the depth profiles of the north and south-facing mid slopes in the four climate regions. Soil samples were collected in biological replicates.

Study site	Depth [cm]	IGSN	Clay* [%]	Sand* [%]	Silt* [%]	pH* ($CaCl_2$)	TOC [%]	C/N	Plant-available P* [mg kg^{-1}]	Total Nitrogen* [%]	BD* [Mg m^{-3}]	Total Sulfur* [-]	Fe_{ox}/Fe_d* [-]
**Pan de Azúcar**													
	0 - 5	*GFRO100UD*	10.0	68.4	21.6	8.10	0.05	2.9	0.44	0.02	1.20	0.10	0.18
AZPED21	5 - 10	*GFRO100UE*	9.1	78.7	12.2	8.12	0.05	3.4	0.27	0.01	1.37	0.44	0.08
(North-facing mid slope)	10 - 20	*GFRO100UF*	18.4	70.7	10.9	8.09	0.05	3.2	1.63	0.02	1.32	2.88	0.10
	20 - 40	*GFRO100UG*	18.5	53.6	27.9	7.96	0.05	3.2	5.50	0.02	n.d.	3.84	0.05
Replicates	0 - 5	*GFLMM00LP*	12.5	67.0	20.5	7.93	0.15	n.d.	n.d.	n.d.	1.23	n.d.	n.d.
	5 - 10	*GFLMM00NW*	n.d.	n.d.	n.d.	n.d.	0.05	n.d.	n.d.	n.d.	n.d.	n.d.	n.d.
	10 - 20	*GFLMM00NX*	n.d.	n.d.	n.d.	n.d.	0.05	n.d.	n.d.	n.d.	n.d.	n.d.	n.d.
	20 - 40	*GFLMM00NY*	n.d.	n.d.	n.d.	n.d.	0.05	n.d.	n.d.	n.d.	n.d.	n.d.	n.d.
**Santa Gracia**													
	0 - 5	*GFRO100VF*	10.3	74.8	14.9	6.21	0.3	7.1	36.61	0.04	1.54	0.02	0.64
SGPED70	5 - 10	*GFRO100VG*	11.4	77.8	10.8	n.d.	0.31	n.d.	37.21	n.d.	1.62	n.d.	n.d.
(North-facing mid slope)	10 - 20	*GFRO100VH*	14.2	74.5	11.4	5.77	0.2	n.d.	n.d.	n.d.	1.65	n.d.	n.d.
	20 - 40	*GFRO100VJ*	20.0	65.5	14.4	n.d.	0.05	n.d.	n.d.	n.d.	1.66	n.d.	n.d.
Replicates	0 - 5	*GFLMM00NS*	12.9	69.9	17.2	n.d.	0.25	n.d.	88.54	n.d.	1.53	n.d.	n.d.
	5 - 10	*GFLMM00NT*	11.3	73.1	15.6	6.22	0.17	4.8	48.30	0.04	1.45	0.03	0.31
	10 - 20	*GFLMM00NU*	23.7	59.9	16.4	n.d.	0.17	n.d.	88.54	n.d.	1.39	n.d.	n.d.
	20 - 40	*GFLMM00NV*	29.8	58.7	11.5	6.00	0.13	4.6	9.76	0.03	n.d.	0.02	0.37
**La Campana**													
	0 - 5	*GFRO1002V*	9.2	72.5	18.3	5.74	2.4	11.0	26.12	0.22	1.30	0.03	0.36
LCPED40	5 - 10	*GFRO1002W*	8.3	72.8	18.9	5.37	1.1	10.2	35.36	0.11	1.35	0.02	0.22
(North-facing mid slope)	10 - 20	*GFRO1002X*	9.5	73.3	17.2	5.06	0.73	15.2	41.27	0.05	1.51	0.03	0.27
	20 - 40	*GFRO1002Y*	12.4	70.2	17.4	4.93	0.47	11.9	21.64	0.04	1.53	0.03	0.19

Replicates	0 - 5	*GFLMM00NA*	9.8	69.4	20.8	6.21	3	16.0	8.34	0.19	1.20	0.04	n.d.
	5 - 10	*GFLMM00NB*	8.4	69.4	22.3	5.91	0.6	n.d.	31.72	n.d.	1.43	n.d.	n.d.
	10 - 20	*GFLMM00NC*	9.0	71.7	19.3	5.76	0.53	n.d.	47.52	n.d.	1.53	n.d.	n.d.
	20 - 40	*GFLMM00ND*	13.6	66.7	19.7	5.13	0.42	n.d.	7.89	n.d.	1.51	n.d.	n.d.
**Nahuelbuta**													
	0 - 5	*GFRO1001R*	19.8	59.7	20.5	4.57	4.7	15.7	3.33	0.30	0.78	0.04	0.82
NAPED40	5 - 10	*GFRO1001S*	20.0	59.1	20.9	4.52	4.4	15.8	2.11	0.28	0.90	0.05	0.92
(North-facing mid slope)	10 - 20	*GFRO1001T*	21.2	54.7	24.1	4.51	2.9	9.9	1.59	0.29	0.92	0.05	0.91
	20 - 40	*GFRO1001U*	22.4	55.3	22.3	4.57	1.2	5.3	1.33	0.22	0.97	0.05	0.99
Replicates	0 - 5	*GFLMM00MU*	27.7	42.3	29.9	4.42	5.6	18.3	2.03	0.31	0.79	0.05	0.96
	5 - 10	*GFLMM00MV*	22.7	51.9	25.3	4.38	4.2	17.9	1.24	0.23	0.77	0.04	0.84
	10 - 20	*GFLMM00MW*	25.4	46.2	28.5	4.34	5.4	18.3	1.06	0.30	0.79	0.04	0.89
	20 - 40	*GFLMM00MX*	27.7	44.1	28.1	4.55	4.5	18.7	0.75	0.24	0.95	0.04	1.01
**Pan de Azúcar**													
	0 - 5	*GFRO100TX*	12.1	64.4	23.6	8.01	0.14	n.d	0.72	n.d	1.19	n.d.	n.d.
AZPED50	5 - 10	*GFRO100TY*	7.5	81.3	11.2	8.1	0.05	5	2.95	0.01	1.59	4.31	0.01
(South-facing mid slope)	10 - 20	*GFRO100TZ*	n.d	n.d	n.d	n.d	0.05	n.d	n.d	n.d	n.d.	n.d.	n.d.
	20 - 40	*GFRO100U0*	n.d	n.d	n.d	n.d	0.05	n.d	n.d	n.d	n.d.	n.d.	n.d.
Replicates	0 - 5	*GFLMM00P7*	12.6	59.4	28.0	8	0.05	n.d	n.d	n.d	1.51	n.d.	n.d.
	5 - 10	*GFLMM00P8*	11.4	70.2	18.4	8.16	0.05	n.d	n.d	n.d	n.d.	n.d.	n.d.
	10 - 20	*GFLMM00P9*	11.4	71.0	17.6	8.25	0.05	2.5	0.19	0.02	n.d.	0.10	0.05
	20 - 40	*GFLMM00PA*	n.d	n.d	n.d	n.d	0.05	n.d	n.d	n.d	n.d.	n.d.	n.d.
**Santa Gracia**													
	0 - 5	*GFRO100UU*	5.6	76.8	17.5	6.28	0.44	8.8	38.02	0.05	1.48	0.03	1.06
SGPED40	5 - 10	*GFRO100UV*	5.8	78.9	15.3	6.29	0.3	10	31.38	0.03	1.55	0.02	0.91
(South-facing mid slope)	10 - 20	*GFRO100V3*	7.5	78.6	13.9	6.18	0.39	13	9.58	0.03	1.55	0.03	0.72
	20 - 40	*GFRO100UW*	8.2	78.1	13.7	6.11	0.23	11.5	6.93	0.02	1.44	0.03	1.03

Replicates	0 - 5	*GFLMM00NJ*	7.3	77.5	15.2	6.41	0.58	8.3	28.22	0.07	1.54	0.03	1.06
	5 - 10	*GFLMM00NK*	7.8	75.4	16.8	6.57	0.5	10	7.04	0.05	1.50	0.02	0.74
	10 - 20	*GFLMM00NL*	6.5	81.5	12.0	6.56	0.19	3.8	6.18	0.05	1.45	0.05	0.65
	20 - 40	*GFLMM00NM*	8.4	77.9	13.7	6.22	0.18	4.5	5.64	0.04	1.50	0.04	0.57
**La Campana**													
	0 - 5	*GFRO10029*	9.7	73.2	17.1	5.55	1.9	5	6.21	0.38	0.64	0.07	0.44
LCPED20	5 - 10	*GFRO1002A*	8.9	74.7	16.5	5.34	1.7	12.1	6.81	0.14	1.04	0.03	0.31
(South-facing mid slope)	10 - 20	*GFRO1002B*	9.5	75.4	15.1	5.31	0.78	9.8	5.36	0.08	1.23	0.02	0.34
	20 - 40	*GFRO1002C*	10.5	75.5	14.1	5.06	0.65	8.1	4.33	0.08	1.25	0.02	0.29
Replicates	0 - 5	*GFLMM00N2*	10.1	70.9	19.0	6.12	5.9	10.9	9.56	0.54	0.87	0.06	0.66
	5 - 10	*GFLMM00N3*	12.0	71.7	16.3	5.97	3.1	6.1	6.84	0.51	0.86	0.05	0.50
	10 - 20	*GFLMM00N4*	11.9	72.9	15.2	5.64	1.7	7.1	3.82	0.24	1.20	0.03	0.41
	20 - 40	*GFLMM00N5*	10.3	74.6	15.1	5.45	1.1	10	4.89	0.11	1.12	0.03	0.45
**Nahuelbuta**													
	0 - 5	*GFRO10019*	24.5	56.0	19.5	4.01	9.6	21.3	1.47	0.45	0.66	0.05	0.96
NAPED20	5 - 10	*GFRO10018*	26.8	53.7	19.5	4.07	8.4	22.7	1.61	0.37	0.72	0.05	1.14
(South-facing mid slope)	10 - 20	*GFRO1001A*	27.6	51.3	21.1	4.15	7.3	22.1	2.61	0.33	0.74	0.07	0.75
	20 - 40	*GFRO1001B*	27.4	51.2	21.4	4.33	4.8	21.8	2.13	0.22	0.77	0.06	0.80
Replicates	0 - 5	*GFLMM00ML*	28.2	47.5	24.3	4.26	6.8	18.9	2.38	0.36	0.69	0.05	0.81
	5 - 10	*GFLMM00MM*	27.0	49.5	23.4	4.25	6.3	19.1	1.94	0.33	0.73	0.05	0.99
	10 - 20	*GFLMM00MN*	27.3	48.1	24.6	4.28	4.4	16.9	1.24	0.26	0.79	0.05	0.01
	20 - 40	*GFLMM00MP*	33.3	44.6	22.1	4.42	3.7	17.6	0.8	0.21	0.75	0.09	0.89

* Bernhard et al. (2018, https://doi.org/10.1016/j.catena.2018.06.018)
n.d. = not determined.

Table S2. List of total amplicon sequence variants (ASVs) per sample. Sequence data was obtained by Illumina HiSeq amplicon sequencing of 16S rRNA gene fragments. Biological duplicates are indicated.

Study site	Depth [cm]	IGSN	ASV reads			
			filtered	merged & orientation corrected		kept [%]
**Pan de Azúcar**						
	0 - 5	*GFRO100UD*	453,688	382,353	363,088	80
AZPED21	5 - 10	*GFRO100UE*	76,688	60,667	60,116	78.4
(North-facing mid	10 - 20	*GFRO100UF*	47,723	39,029	38,988	81.7
slope)	20 - 40	*GFRO100UG*	66,472	51,251	51,225	77.1
Replicates	0 - 5	*GFLMM00LP*	530,363	444,676	408,694	77.1
	5 - 10	*GFLMM00NW*	133,561	114,613	113,475	85
	10 - 20	*GFLMM00NX*	53,535	41,450	41,376	77.3
	20 - 40	*GFLMM00NY*	112,257	92,337	91,441	81.5
**Santa Gracia**						
	0 - 5	*GFRO100VF*	465,726	397,870	382,245	82.1
SGPED70	5 - 10	*GFRO100VG*	69,582	48,431	48,255	69.3
(North-facing mid	10 - 20	*GFRO100VH*	105,326	78,781	78,631	74.7
slope)	20 - 40	*GFRO100VJ*	176,644	135,840	135,231	76.6
Replicates	0 - 5	*GFLMM00NS*	413,582	354,139	336,095	81.3
	5 - 10	*GFLMM00NT*	116,196	87,599	87,268	75.1
	10 - 20	*GFLMM00NU*	160,225	121,536	121,163	75.6
	20 - 40	*GFLMM00NV*	92,040	67,921	67,555	73.4
**La Campana**						
	0 - 5	*GFRO1002V*	55,263	33,699	33,689	61
LCPED40	5 - 10	*GFRO1002W*	196,807	147,923	147,647	75
(North-facing mid	10 - 20	*GFRO1002X*	310,925	236,636	235,973	75.9
slope)	20 - 40	*GFRO1002Y*	340,144	267,038	264,541	77.8
Replicates	0 - 5	*GFLMM00NA*	427,504	328,626	325,180	76.1
	5 - 10	*GFLMM00NB*	218,074	153,877	152,818	70.1
	10 - 20	*GFLMM00NC*	353,118	265,790	264,990	75
	20 - 40	*GFLMM00ND*	352,435	270,419	263,524	74.8

**Nahuelbuta**						
	0 - 5	*GFRO1001R*	397,206	334,150	314,133	79.1
NAPED40	5 - 10	*GFRO1001S*	106,925	79,046	77,277	72.3
(North-facing mid	10 - 20	*GFRO1001T*	188,950	146,157	142,210	75.3
slope)	20 - 40	*GFRO1001U*	203,695	157,859	153,892	75.6
Replicates	0 - 5	*GFLMM00MU*	146,180	105,252	103,542	70.8
	5 - 10	*GFLMM00MV*	89,729	67,825	66,885	74.5
	10 - 20	*GFLMM00MW*	212,668	177,132	172,813	81.3
	20 - 40	*GFLMM00MX*	295,469	243,261	233,672	79.1
**Pan de Azúcar**						
	0 - 5	*GFLMM00B0*	378,070	297,364	281,586	74.5
AZPED50	5 - 10	*GFLMM00AJ*	103,190	88,431	86,980	84.3
(South-facing mid	10 - 20	*GFLMM00AH*	494,245	447,348	405,476	82
slope)	20 - 40	*GFLMM00AG*	71,162	59,594	59,004	82.9
Replicates	0 - 5	*GFLMM00AF*	409,029	369,969	367,185	89.8
	5 - 10	*GFLMM00AE*	342,184	163,909	157,953	46.2
	10 - 20	*GFLMM00AD*	410,477	368,846	354,279	86.3
	20 - 40	*GFLMM00AC*	55,222	428,38	42,612	77.2
**Santa Gracia**						
	0 - 5	*GFLMM00BH*	280,167	253,973	250,769	89.5
SGPED40	5 - 10	*GFLMM00BJ*	294,057	262,572	259,692	88.3
(South-facing mid	10 - 20	*GFLMM00CU*	434,551	392,896	386,827	89
slope)	20 - 40	*GFLMM00CW*	85,040	56,484	56,420	66.3
Replicates	0 - 5	*GFLMM009W*	558,578	489,569	479,285	85.8
	5 - 10	*GFLMM009V*	433,238	396,073	391,221	90.3
	10 - 20	*GFLMM009U*	329,705	245,764	243,910	74
	20 - 40	*GFLMM009T*	96,382	66,604	66,150	68.6
**La Campana**						
	0 - 5	*GFLMM00CJ*	561,819	504,263	497,776	88.6
LCPED20	5 - 10	*GFLMM00B4*	315,879	275,404	274,034	86.8
	10 - 20	*GFLMM00CH*	317,624	283,406	281,281	88.6

(South-facing mid slope)	20 - 40	*GFLMM00B3*	177,313	130,598	129,995	73.3
Replicates	0 - 5	*GFLMM00A3*	638,016	562,127	554,849	87
	5 - 10	*GFLMM00A2*	231,307	194,100	191,685	82.9
	10 - 20	*GFLMM00A1*	650,913	588,247	580,984	89.3
	20 - 40	*GFLMM00A0*	112,264	790,79	78,773	70.2
**Nahuelbuta**						
	0 - 5	*GFLMM00BV*	495,783	390,285	364,764	73.6
NAPED20	5 - 10	*GFLMM00BW*	525,175	470,781	463,539	88.3
(South-facing mid	10 - 20	*GFLMM00BU*	564,367	503,588	491,555	87.1
slope)	20 - 40	*GFLMM00BT*	186,354	148,884	146,770	78.8
Replicates	0 - 5	*GFLMM001T*	683,194	612,307	598,851	87.7
	5 - 10	*GFLMM001S*	735,811	647,192	618,336	84
	10 - 20	*GFLMM001R*	331,133	291,847	275,445	83.2
	20 - 40	*GFLMM001Q*	170,633	138,681	137,590	80.6

Table S3. Pearson correlation of soil bacterial diversity and physicochemical parameters in the north and south-facing mid slope.

Slope aspect	Property	Sand	Silt	Clay	BD	pH	Plant-available P	TOC	Nt	St	C/N	Fe_{ox}/Fe_d	Richness	Shannon (H)	Evenness
	Sand		2.95E-08	5.12E-10	1.89E-06	0.00796	0.03233	2.36E-05	0.00010	0.54284	0.01119	7.50E-05	0.24233	0.33734	0.24172
	Silt	-0.8042		0.03315	6.75E-07	0.03079	0.04956	0.00012	0.00049	0.75470	0.00414	0.00681	0.84395	0.93969	0.35705
	Clay	-0.8540	0.3775		0.00452	0.03055	0.11598	0.00347	0.00476	0.49541	0.13927	0.00031	0.04148	0.15003	0.31231
	BD	0.7493	-0.7694	-0.5040		0.01612	0.00175	1.18E-09	2.73E-09	0.78272	0.00042	1.05E-05	0.16224	0.21171	0.28541
	pH	0.4608	-0.3824	-0.3829	0.4356		0.59326	1.57E-05	1.29E-06	0.00039	6.98E-07	3.75E-07	0.02715	0.00056	0.00262
	Plant-available P	0.3792	-0.3500	-0.2834	0.5473	-0.0981		0.04927	0.11257	0.17384	0.67036	0.57352	0.03349	0.01460	0.05113
North-facing slope	TOC	-0.6738	0.6269	0.5013	-0.8596	-0.6844	-0.3504		1.12E-14	0.09396	1.02E-09	1.03E-07	0.86932	0.42626	0.34789
	N_t	-0.6501	0.5972	0.5014	-0.8656	-0.7570	-0.2958	0.9408		0.05597	1.29E-06	2.44E-09	0.90706	0.32503	0.21899
	S_t	-0.1156	0.0595	0.1294	0.0546	0.6060	-0.2550	-0.3114	-0.3526		0.00948	0.01259	0.00148	7.86E-07	0.00017
	C/N	-0.4567	0.5082	0.2764	-0.6213	-0.7687	-0.0810	0.8611	0.7569	-0.4658		0.00014	0.03357	0.00802	0.02263
	Fe_{ox}/Fe_d	-0.6590	0.4834	0.6141	-0.7299	-0.7800	-0.1070	0.8015	0.8515	-0.4500	0.6401		0.79906	0.22861	0.26842
	Richness	0.2128	0.0362	-0.3625	0.2618	-0.3904	0.3769	0.0303	0.0223	-0.5544	0.3891	0.0485		3.97E-12	0.00095
	Shannon (H)	0.1753	-0.0139	-0.2604	0.2348	-0.5764	0.4278	0.1457	0.1860	-0.7665	0.4748	0.2266	0.8965		6.38E-09
	Evenness	0.2130	-0.1683	-0.1844	0.2016	-0.5140	0.3478	0.1715	0.2312	-0.6340	0.4149	0.2087	0.5563	0.8249	
	Sand		3.33E-09	3.20E-18	4.49E-06	0.00085	0.0139	1.57E-06	0.00156	0.3899	1.86E-07	0.39085	0.86394	0.90989	0.63512
	Silt	-0.8479		2.88E-05	0.00496	0.22103	0.0986	0.00704	0.00197	0.3084	4.81E-05	0.63610	0.93116	0.59250	0.38461
	Clay	-0.9673	0.6858		9.03E-07	3.45E-05	0.0100	9.21E-08	0.00249	0.4500	8.16E-08	0.33046	0.78489	0.92488	0.80836
	BD	0.7492	-0.5158	-0.7817		6.74E-07	0.0203	1.54E-08	1.35E-08	0.0629	1.39E-04	0.73354	0.02080	0.09278	0.68480
	pH	0.5768	-0.2302	-0.6809	0.7871		0.6597	1.03E-06	0.00055	0.0055	1.22E-08	0.07492	0.00456	0.00020	0.00174
	Plant-available P	0.4442	-0.3073	-0.4629	0.4362	0.0838		0.14337	0.18551	0.4796	0.33048	0.03535	0.19607	0.05745	0.05765
South-facing slope	TOC	-0.7530	0.4817	0.8032	-0.8451	-0.7613	-0.2737		4.98E-08	0.2306	2.56E-08	0.07286	0.00500	0.04980	0.28518
	N_t	-0.5695	0.5593	0.5489	-0.8635	-0.6110	-0.2577	0.8292		0.1531	5.51E-03	0.45461	0.00012	0.02064	0.17763
	S_t	0.1690	-0.1996	-0.1488	0.3700	0.5105	-0.1393	-0.2341	-0.2773		0.15602	0.12133	0.01771	0.01075	0.10605
	C/N	-0.8092	0.6903	0.8220	-0.6784	-0.8480	-0.1909	0.8384	0.5105	-0.2754		0.00906	0.15476	6.01E-02	0.11351
	Fe_{ox}/Fe_d	-0.1756	0.0974	0.1987	0.0733	-0.3553	0.4143	0.3576	0.1533	-0.3115	0.5014		0.22820	0.01653	0.01255
	Richness	-0.0327	-0.0165	0.0520	-0.4347	-0.5035	0.2428	0.4841	0.6637	-0.4448	0.2762	0.2447		7.32E-09	0.00033
	Shannon (H)	0.0216	-0.1018	0.0180	-0.3238	-0.6285	0.3507	0.3497	0.4352	-0.4744	0.3597	0.4656	0.8232		1.22E-14
	Evenness	0.0903	-0.1647	-0.0462	-0.0802	-0.5476	0.3504	0.1949	0.2622	-0.3120	0.3058	0.4825	0.5945	0.9307	

Table S4. Relative abundances in % of dominant bacterial phyla obtained in the four climate regions and four depths. Community composition was studied in biological duplicates, based on Illumina HiSeq amplicon sequencing.

Study site	Depth [cm]	IGSN	*Acidobacteria*	*Actinobacteria*	*Chloroflexi*	*Gemmatimonadetes* [Relative abundance %]	*Planctomycetes*	*Proteobacteria*	*Verrucomicrobia*
**Pan de Azúcar**									
	0 - 5	GFRO100UD	1.8	54.1	12.0	6.1	1.5	16.7	1.1
AZPED21	5 - 10	GFRO100UE	2.9	51.3	5.4	8.1	3.1	17.4	1.4
(North-facing mid	10 - 20	GFRO100UF	1.0	41.3	3.2	12.1	4.4	17.1	0.7
slope)	20 - 40	GFRO100UG	2.4	46.0	4.5	7.2	1.1	15.9	0.7
Replicates	0 - 5	GFLMM00LP	7.3	32.1	18.7	3.4	9.2	10.8	7.5
	5 - 10	GFLMM00NW	2.8	55.0	9.3	10.0	1.2	13.7	0.6
	10 - 20	GFLMM00NX	1.9	62.2	8.2	12.5	0.4	9.9	0.6
	20 - 40	GFLMM00NY	5.2	37.3	6.6	1.2	4.1	21.8	3.7
**Santa Gracia**									
	0 - 5	GFRO100VF	8.0	42.5	11.0	5.0	5.9	17.1	2.8
SGPED70	5 - 10	GFRO100VG	14.2	29.3	13.2	3.4	6.8	17.7	7.7
(North-facing mid	10 - 20	GFRO100VH	8.6	40.8	9.1	4.6	3.7	19.2	4.8
slope)	20 - 40	GFRO100VJ	10.3	42.6	10.2	2.8	4.7	13.1	7.2
Replicates	0 - 5	GFLMM00NS	3.5	27.2	9.8	4.0	3.8	29.5	2.4
	5 - 10	GFLMM00NT	9.9	36.2	10.2	10.6	1.7	19.4	3.0
	10 - 20	GFLMM00NU	18.7	27.3	9.2	4.3	6.7	14.5	10.9
	20 - 40	GFLMM00NV	11.7	35.3	10.7	2.3	6.1	11.6	13.0
**La Campana**									
	0 - 5	GFRO1002V	16.0	23.8	5.1	1.8	6.3	33.9	5.2
LCPED40	5 - 10	GFRO1002W	13.8	27.1	6.8	3.5	5.2	29.0	5.4
(North-facing mid	10 - 20	GFRO1002X	18.4	23.9	9.1	3.3	8.4	20.4	7.8
slope)	20 - 40	GFRO1002Y	17.7	24.1	8.5	3.7	7.6	18.3	10.9
Replicates	0 - 5	GFLMM00NA	18.4	19.2	7.9	2.2	13.6	25.4	5.0
	5 - 10	GFLMM00NB	14.8	21.7	8.6	4.5	6.5	27.9	6.5
	10 - 20	GFLMM00NC	18.4	18.8	10.7	3.1	9.9	20.5	10.0
	20 - 40	GFLMM00ND	17.3	21.5	8.5	4.8	7.9	16.2	12.4
**Nahuelbuta**									
	0 - 5	GFRO1001R	14.7	13.9	13.1	1.1	10.0	26.7	17.5
NAPED40	5 - 10	GFRO1001S	12.2	14.9	17.5	1.0	4.5	33.2	11.9
	10 - 20	GFRO1001T	15.6	9.1	25.3	0.7	7.3	22.7	14.6

(North-facing mid slope)	20 - 40	*GFRO1001U*	16.8	5.4	38.2	1.6	3.4	13.2	7.8
Replicates	0 - 5	*GFLMM00MU*	16.0	9.1	21.0	1.4	11.4	23.6	7.8
	5 - 10	*GFLMM00MV*	19.3	6.7	19.6	0.6	13.8	25.6	9.1
	10 - 20	*GFLMM00MW*	16.6	10.3	17.8	0.9	5.8	36.5	6.9
	20 - 40	*GFLMM00MX*	13.8	12.6	20.2	1.3	8.0	30.2	9.0
**Pan de Azúcar**									
	0 - 5	*GFLMM00B0*	1.5	33.5	14.9	9.4	13.8	15.5	0.7
AZPED50	5 - 10	*GFLMM00AJ*	1.2	54.4	2.6	15.1	6.1	8.0	0.5
(South-facing mid	10 - 20	*GFLMM00AH*	0.9	72.8	0.9	3.3	2.0	8.5	0.5
slope)	20 - 40	*GFLMM00AG*	1.1	35.3	5.7	11.4	7.2	24.0	0.4
Replicates	0 - 5	*GFLMM00AF*	1.0	46.1	12.7	21.2	2.4	9.0	0.1
	5 - 10	*GFLMM00AE*	0.8	54.8	3.9	14.8	4.9	10.5	0.8
	10 - 20	*GFLMM00AD*	1.0	73.2	1.4	3.6	0.3	10.5	0.2
	20 - 40	*GFLMM00AC*	1.7	27.8	0.3	0.5	1.9	34.7	0.6
**Santa Gracia**									
	0 - 5	*GFLMM00BH*	11.9	37.0	6.4	3.5	7.7	21.0	3.2
SGPED40	5 - 10	*GFLMM00BJ*	9.7	38.8	7.9	5.7	2.6	23.3	3.1
(South-facing mid	10 - 20	*GFLMM00CU*	19.5	32.5	6.8	3.4	8.2	16.3	7.5
slope)	20 - 40	*GFLMM00CW*	20.6	31.8	7.5	2.6	5.5	12.4	13.1
Replicates	0 - 5	*GFLMM009W*	7.2	31.0	5.8	4.4	3.3	25.6	2.1
	5 - 10	*GFLMM009V*	22.9	32.8	6.3	3.1	6.0	12.6	8.6
	10 - 20	*GFLMM009U*	18.5	37.3	7.0	3.2	6.0	11.5	8.5
	20 - 40	*GFLMM009T*	18.7	43.3	3.6	2.6	4.7	14.2	7.6
**La Campana**									
	0 - 5	*GFLMM00CJ*	23.4	20.8	5.2	2.6	12.0	19.2	9.7
LCPED20	5 - 10	*GFLMM00B4*	18.0	28.9	5.9	1.7	5.3	23.5	8.0
(South-facing mid	10 - 20	*GFLMM00CH*	14.7	25.1	10.2	4.8	4.5	21.5	11.3
slope)	20 - 40	*GFLMM00B3*	17.7	19.7	6.3	4.9	6.2	18.6	16.7
Replicates	0 - 5	*GFLMM00A3*	20.2	29.3	4.0	2.5	9.1	21.5	7.4
	5 - 10	*GFLMM00A2*	11.3	37.1	5.0	4.0	3.0	24.3	6.5
	10 - 20	*GFLMM00A1*	20.4	24.8	6.0	4.1	7.3	16.3	12.0
	20 - 40	*GFLMM00A0*	21.3	21.1	6.7	5.7	6.3	17.0	11.5
**Nahuelbuta**									
	0 - 5	*GFLMM00BV*	20.5	18.7	4.0	0.7	10.3	33.0	7.9

NAPED20	5 - 10	*GFLMM00BW*	26.8	13.5	5.7	1.0	10.4	26.5	11.4
(South-facing mid slope)	10 - 20	*GFLMM00BU*	29.2	11.5	11.0	0.6	5.9	32.1	6.0
	20 - 40	*GFLMM00BT*	20.3	10.7	13.4	1.0	9.2	29.3	11.9
Replicates	0 - 5	*GFLMM001T*	21.9	18.6	9.2	1.5	7.5	30.4	5.9
	5 - 10	*GFLMM001S*	22.4	15.6	13.8	0.7	9.4	28.8	6.1
	10 - 20	*GFLMM001R*	18.3	16.6	16.0	0.7	3.5	37.5	4.3
	20 - 40	*GFLMM001Q*	11.6	16.0	18.5	3.1	3.1	37.0	3.3

Table S5. Results of PERMANOVA analysis using adonis function to compare bacterial community composition between sites and slope aspects.

	Df	SumsOfSqs	R2	F	Pr(>F)
Site	3	6.8837	0.27332	7.5223	0.001***
Slope aspect	7	9.5636	0.37972	4.8974	0.001***

Signif. codes: 0 '***' 0.001 '**' 0.01 '*' 0.05 '.' 0.1 ' ' 1

Table S6. Results of PERMANOVA analysis using pairwise adonis test based on Bray-Curtis distances with 999 permutations for comparing bacterial community composition between sites and slope aspects (north and south-facing mid slope).

Comparison	Pairs	Df	SumsOfSqs	F.model	R2	p.value	p.adjusted	sig
	AZ vs SG	1	1.743065	4.694124	0.1353002	0.001	0.006	*
	AZ vs LC	1	2.09087	6.098381	0.1689378	0.001	0.006	*
	AZ vs NA	1	2.591955	8.361754	0.2179711	0.001	0.006	*
	SG vs LC	1	1.268199	4.225949	0.1234721	0.001	0.006	*
	SG vs NA	1	3.113795	11.6526	0.2797568	0.001	0.006	*
Site	LC vs NA	1	2.95958	12.396332	0.2923916	0.001	0.006	*
	AZ-NFS vs AZ-SFS	1	0.6696527	1.691756	0.1078118	0.013	0.364	
	SG-NFS vs SG-SFS	1	0.6604451	2.16635	0.1340037	0.006	0.168	
	LC-NFS vs LC-SFS	1	0.6943934	2.876193	0.170429	0.001	0.028	*
Slope aspect	NA-NFS vs NA-SFS	1	0.6553783	3.771761	0.2122334	0.001	0.028	*

Signif. codes: 0 '***' 0.001 '**' 0.01 '*' 0.05 '.' 0.1 ' ' 1

Table S7. Indicator-value analysis (or IndVal) of bacterial specialists identified in the north and south-facing mid slope. D represents 4 depth profiles – corresponding to D1 (0–5 cm), D2 (5–10 cm), D3 (10–20 cm), and D4 (20–40 cm). Taxa are shown on the level of (c) class, (o) order, (f) family, and (g) genus.

Slope aspect	ASV	Taxa	Site/depth	Indicator value	*p*-value
North-facing slope	ASV_00237	*(p) Acidobacteria (c) Subgroup 6*	LC	0.891061	0.001
	ASV_00278	*(p) Acidobacteria (c) Subgroup 6*	NA	0.978308	0.001
	ASV_00048	*(p) Acidobacteria (g) Candidatus Solibacter*	NA	0.860215	0.001
	ASV_00104	*(p) Acidobacteria (g) RB41*	LC	0.905058	0.001
	ASV_00162	*(p) Acidobacteria (o) Acidobacteriales*	NA	0.918919	0.001
	ASV_00223	*(p) Acidobacteria (o) Acidobacteriales*	NA	0.978041	0.001
	ASV_00286	*(p) Acidobacteria (o) Acidobacteriales*	NA	0.928863	0.001
	ASV_00035	*(p) Acidobacteria (o) Subgroup 2*	NA	0.881072	0.001
	ASV_00090	*(p) Acidobacteria (o) Subgroup 2*	NA	0.875321	0.001
	ASV_00629	*(p) Acidobacteria (o) Subgroup 7*	NA	0.882803	0.001
	ASV_01767	*(p) Acidobacteria (o) Subgroup 7*	AZ-D4	0.966972	0.036
	ASV_01273	*(p) Actinobacteria (c) Acidimicrobiia*	SG	0.875912	0.001
	ASV_00073	*(p) Actinobacteria (f) 67-14*	AZ-D1	0.995273	0.031
	ASV_00133	*(p) Actinobacteria (f) 67-14*	AZ-D1	0.936084	0.029
	ASV_00179	*(p) Actinobacteria (f) 67-14*	AZ-D1	0.971496	0.035
	ASV_00228	*(p) Actinobacteria (f) 67-14*	LC	0.931961	0.001
	ASV_00168	*(p) Actinobacteria (f) Nitriliruptoraceae*	AZ	0.814757	0.005
	ASV_00394	*(p) Actinobacteria (g) Amycolatopsis*	SG	0.858166	0.001
	ASV_00087	*(p) Actinobacteria (g) Conexibacter*	NA	0.908696	0.001
	ASV_00263	*(p) Actinobacteria (g) Conexibacter*	AZ-D1	0.981436	0.033
	ASV_00016	*(p) Actinobacteria (g) Cutibacterium*	AZ	0.803121	0.042
	ASV_00342	*(p) Actinobacteria (g) Gaiella*	LC	0.83559	0.001
	ASV_00349	*(p) Actinobacteria (g) Mycobacterium*	NA	0.958801	0.001
	ASV_00346	*(p) Actinobacteria (g) Rubrobacter*	AZ-D1	0.949301	0.025

ASV_01240	*(p) Actinobacteria (g) Rubrobacter*	SG	1	0.001
ASV_00783	*(p) Actinobacteria (o) Gaiellales*	SG	0.861111	0.001
ASV_00078	*(p) Actinobacteria (o) IMCC26256*	NA	0.900643	0.001
ASV_00009	*(p) Chloroflexi (c) AD3*	NA	0.81335	0.001
ASV_00049	*(p) Chloroflexi (c) AD3*	NA	0.882872	0.001
ASV_00128	*(p) Chloroflexi (c) AD3*	NA	0.969988	0.001
ASV_00387	*(p) Chloroflexi (c) AD3*	NA	0.885714	0.001
ASV_00481	*(p) Chloroflexi (c) AD3*	NA	0.830674	0.001
ASV_00207	*(p) Chloroflexi (c) TK10*	NA	0.978667	0.001
ASV_00234	*(p) Chloroflexi (c) TK10*	NA	0.963082	0.001
ASV_00127	*(p) Chloroflexi (f) JG30-KF-CM45*	AZ-D1	0.930275	0.032
ASV_00294	*(p) Chloroflexi (g) HSB OF53-F07*	NA	0.924911	0.001
ASV_00365	*(p) Chloroflexi (g) HSB OF53-F07*	NA	0.979742	0.001
ASV_00401	*(p) Chloroflexi (g) JG30a-KF-32*	NA	0.907552	0.001
ASV_00941	*(p) Chloroflexi (g) JG30a-KF-32*	NA	0.96875	0.001
ASV_00196	*(p) Chloroflexi (o) C0119*	SG	0.851693	0.002
ASV_03747	*(p) Firmicutes (g) Bacillus*	AZ-D3	0.970817	0.03
ASV_00007	*(p) Firmicutes (g) Paenibacillus*	AZ	0.860612	0.002
ASV_00021	*(p) Firmicutes (g) Staphylococcus*	AZ	0.834679	0.001
ASV_00447	*(p) GAL15 (p) GAL15*	NA	0.817383	0.004
ASV_00416	*(p) Gemmatimonadetes (c) BD2-11 terrestrial group*	AZ	0.831886	0.004
ASV_01253	*(p) Gemmatimonadetes (c) BD2-11 terrestrial group*	AZ-3	0.865734	0.039
ASV_00121	*(p) Planctomycetes (f) Gemmataceae*	NA	0.830455	0.001
ASV_00366	*(p) Planctomycetes (f) Gemmataceae*	NA	0.95734	0.001
ASV_00082	*(p) Proteobacteria (f) A21b*	NA	0.836321	0.001
ASV_00903	*(p) Proteobacteria (f) Rhizobiaceae*	AZ-D4	0.954098	0.037
ASV_00041	*(p) Proteobacteria (f) Xanthobacteraceae*	NA	0.871116	0.001

	ASV_00043	*(p) Proteobacteria (f) Xanthobacteraceae*	NA	0.802617	0.001
	ASV_00124	*(p) Proteobacteria (g) Acidibacter*	NA	0.81672	0.001
	ASV_00221	*(p) Proteobacteria (g) Methylobacterium*	SG	0.811614	0.001
	ASV_00295	*(p) Proteobacteria (g) Rhodomicrobium*	NA	0.875433	0.001
	ASV_00150	*(p) Proteobacteria (g) Sphingomonas*	AZ	0.918938	0.001
	ASV_00034	*(p) Proteobacteria (o) Elsterales*	NA	0.833123	0.001
	ASV_00255	*(p) Proteobacteria (o) Elsterales*	NA	0.839857	0.001
	ASV_00703	*(p) Proteobacteria (o) Elsterales*	NA	0.875	0.001
	ASV_00044	*(p) Proteobacteria (o) WD260*	NA	0.909756	0.001
	ASV_00089	*(p) Rokubacteria (o) Rokubacteriales*	NA	0.878492	0.001
	ASV_00195	*(p) Rokubacteria (o) Rokubacteriales*	LC-D4	0.807018	0.033
	ASV_00302	*(p) Rokubacteria (o) Rokubacteriales*	NA	0.815276	0.001
	ASV_00188	*(p) Verrucomicrobia (g) Candidatus* Udaeobacter	AZ-D1	0.992701	0.028
	ASV_00247	*(p) Verrucomicrobia (g) Candidatus* Udaeobacter	NA	0.809971	0.001
	ASV_00406	*(p) Verrucomicrobia (g) Candidatus* Udaeobacter	NA	0.873901	0.001
	ASV_01663	*(p) Verrucomicrobia (g) Candidatus* Udaeobacter	SG	1	0.001
	ASV_00024	*(p) Verrucomicrobia (g) Candidatus Xiphinematobacter*	NA	0.802878	0.001
	ASV_00137	*(p) Verrucomicrobia (g) Candidatus Xiphinematobacter*	NA	0.982554	0.001
	ASV_00293	*(p) Verrucomicrobia (g) Candidatus Xiphinematobacter*	NA	0.996894	0.001
	ASV_00482	*(p) Verrucomicrobia (g) Candidatus Xiphinematobacter*	NA	0.946367	0.002
	ASV_00494	*(p) Verrucomicrobia (g) Candidatus Xiphinematobacter*	NA	0.825428	0.001
	ASV_00545	*(p) Verrucomicrobia (g) Candidatus Xiphinematobacter*	NA	0.953912	0.001
South-facing slope	ASV_00278	*(p) Acidobacteria; (c) Subgroup 6*	NA	1.000	0.001
	ASV_00096	*(p) Acidobacteria; (g) Bryobacter*	NA	0.857	0.001
	ASV_00252	*(p) Acidobacteria; (g) Bryobacter*	NA	0.950	0.001
	ASV_00048	*(p) Acidobacteria; (g) Candidatus Solibacter*	NA	0.889	0.001
	ASV_00235	*(p) Acidobacteria; (g) Candidatus Solibacter*	NA	0.927	0.001

ASV_00027	*(p) Acidobacteria; (g) Granulicella*	NA	0.932	0.001
ASV_00028	*(p) Acidobacteria; (g) Granulicella*	NA	0.855	0.001
ASV_00060	*(p) Acidobacteria; (g) Granulicella*	NA	0.848	0.001
ASV_00011	*(p) Acidobacteria; (g) RB41*	SG	0.814	0.001
ASV_00116	*(p) Acidobacteria; (g) RB41*	SG	0.862	0.001
ASV_00039	*(p) Acidobacteria; (o) Acidobacteriales*	NA	0.918	0.001
ASV_00072	*(p) Acidobacteria; (o) Acidobacteriales*	NA	0.868	0.001
ASV_00162	*(p) Acidobacteria; (o) Acidobacteriales*	NA	0.916	0.001
ASV_00035	*(p) Acidobacteria; (o) Subgroup 2*	NA	0.946	0.001
ASV_00058	*(p) Acidobacteria; (o) Subgroup 2*	NA	0.862	0.001
ASV_00090	*(p) Acidobacteria; (o) Subgroup 2*	NA	0.996	0.001
ASV_00123	*(p) Acidobacteria; (o) Subgroup 2*	NA	0.965	0.001
ASV_00140	*(p) Acidobacteria; (o) Subgroup 2*	NA	0.941	0.001
ASV_00222	*(p) Acidobacteria; (o) Subgroup 2*	NA	0.952	0.001
ASV_00280	*(p) Acidobacteria; (o) Subgroup 2*	NA	0.859	0.001
ASV_00320	*(p) Acidobacteria; (o) Subgroup 2*	NA	0.995	0.001
ASV_00148	*(p) Actinobacteria; (c) Acidimicrobiia*	NA	0.937	0.001
ASV_00175	*(p) Actinobacteria; (c) Acidimicrobiia*	NA	0.946	0.001
ASV_00268	*(p) Actinobacteria; (c) Acidimicrobiia*	AZ- D1	0.974	0.035
ASV_00292	*(p) Actinobacteria; (f) 67-14*	LC	0.845	0.001
ASV_00208	*(p) Actinobacteria; (f) 67-14*	SG	0.823	0.001
ASV_00037	*(p) Actinobacteria; (f) 67-14*	AZ-D1	0.821	0.028
ASV_00385	*(p) Actinobacteria; (f) Euzebyaceae*	AZ-D2	0.882	0.045
ASV_00627	*(p) Actinobacteria; (f) Nitriliruptoraceae*	AZ-D2	0.981	0.033
ASV_00113	*(p) Actinobacteria; (f) Thermomonosporaceae*	NA	0.952	0.002
ASV_00002	*(p) Actinobacteria; (f) Thermomonosporaceae*	AZ-D3	0.865	0.033
ASV_00272	*(p) Actinobacteria; (g) Acidothermus*	NA	0.933	0.001
ASV_00283	*(p) Actinobacteria; (g) Acidothermus*	NA	0.930	0.001

ASV_00087	*(p) Actinobacteria; (g) Conexibacter*	NA	0.901	0.001
ASV_00010	*(p) Actinobacteria; (g) Conexibacter*	AZ-D1	0.835	0.039
ASV_00066	*(p) Actinobacteria; (g) Conexibacter*	AZ	0.813	0.017
ASV_00337	*(p) Actinobacteria; (g) Conexibacter*	AZ-D2	0.980	0.025
ASV_00497	*(p) Actinobacteria; (g) Conexibacter*	AZ-D1	0.906	0.043
ASV_00016	*(p) Actinobacteria; (g) Cutibacterium*	AZ	0.859	0.001
ASV_00166	*(p) Actinobacteria; (g) Rubrobacter*	SG	0.801	0.001
ASV_00180	*(p) Actinobacteria; (g) Rubrobacter*	AZ-D3	0.835	0.029
ASV_00386	*(p) Actinobacteria; (o) Gaiellales*	LC	0.983	0.001
ASV_00078	*(p) Actinobacteria; (o) IMCC26256*	NA	0.913	0.001
ASV_00253	*(p) Actinobacteria; (o) IMCC26256*	NA	0.883	0.001
ASV_00187	*(p) Actinobacteria; (p) Actinobacteria*	AZ-D2	0.871	0.026
ASV_00057	*(p) Bacteroidetes; (f) Chitinophagaceae*	NA	0.908	0.001
ASV_00420	*(p) Bacteroidetes; (f) Cyclobacteriaceae*	AZ	0.842	0.002
ASV_00229	*(p) Bacteroidetes; (f) Rhodothermaceae*	AZ-D1	0.863	0.045
ASV_01688	*(p) Bacteroidetes; (f) Rhodothermaceae*	AZ-D2	0.990	0.029
ASV_00215	*(p) Bacteroidetes; (g) Segetibacter*	SG-D1	0.915	0.026
ASV_00009	*(p) Chloroflexi; (c) AD3*	NA	0.877	0.001
ASV_00049	*(p) Chloroflexi; (c) AD3*	NA	0.866	0.001
ASV_00128	*(p) Chloroflexi; (c) AD3*	NA	0.985	0.001
ASV_00158	*(p) Chloroflexi; (c) KD4-96*	LC	0.824	0.001
ASV_00234	*(p) Chloroflexi; (c) TK10*	NA	0.969	0.001
ASV_00359	*(p) Chloroflexi; (c) TK10*	NA	0.855	0.001
ASV_00856	*(p) Chloroflexi; (f) AKYG1722*	AZ-D2	0.847	0.031
ASV_00093	*(p) Chloroflexi; (f) Caldilineaceae*	AZ-D1	0.977	0.036
ASV_00194	*(p) Chloroflexi; (f) Ktedonobacteraceae*	NA	0.907	0.001
ASV_00139	*(p) Chloroflexi; (g) HSB OF53-F07*	NA	0.893	0.001
ASV_00231	*(p) Chloroflexi; (g) HSB OF53-F07*	NA	0.967	0.001

ASV_00111	*(p) Firmicutes; (g) Anoxybacillus*	AZ-D4	0.937	0.024
ASV_00021	*(p) Firmicutes; (g) Staphylococcus*	AZ	0.827	0.004
ASV_00520	*(p) Gemmatimonadetes; (c) BD2-11 terrestrial group*	AZ-D2	0.922	0.035
ASV_00109	*(p) Gemmatimonadetes; (c) S0134 terrestrial group*	AZ-D2	0.858	0.037
ASV_00454	*(p) Gemmatimonadetes; (c) S0134 terrestrial group*	AZ-D2	0.990	0.031
ASV_00178	*(p) Gemmatimonadetes; (f) Gemmatimonadaceae*	LC	0.957	0.001
ASV_00241	*(p) Gemmatimonadetes; (f) Gemmatimonadaceae*	LC	0.971	0.001
ASV_00186	*(p) Gemmatimonadetes; (f) Longimicrobiaceae*	AZ-D1	0.975	0.036
ASV_00264	*(p) Gemmatimonadetes; (f) Longimicrobiaceae*	AZ-D1	0.956	0.034
ASV_00121	*(p) Planctomycetes; (f) Gemmataceae*	NA	0.911	0.001
ASV_01153	*(p) Planctomycetes; (g) Rhodopirellula*	AZ-D2	0.944	0.037
ASV_00160	*(p) Planctomycetes; (g) Singulisphaera*	NA	0.904	0.001
ASV_00271	*(p) Planctomycetes; (o) Planctomycetales*	NA	0.876	0.001
ASV_00206	*(p) Proteobacteria; (c) Alphaproteobacteria*	NA	0.898	0.001
ASV_00082	*(p) Proteobacteria; (f) A21b*	NA	0.881	0.001
ASV_00041	*(p) Proteobacteria; (f) Xanthobacteraceae*	NA	0.958	0.001
ASV_00059	*(p) Proteobacteria; (f) Xanthobacteraceae*	NA	0.929	0.001
ASV_00114	*(p) Proteobacteria; (f) Xanthobacteraceae*	NA	0.959	0.001
ASV_00316	*(p) Proteobacteria; (f) Xanthobacteraceae*	NA	0.988	0.001
ASV_00163	*(p) Proteobacteria; (g) Acidibacter*	NA	0.921	0.001
ASV_00282	*(p) Proteobacteria; (g) Burkholderia-Caballeronia-Paraburkholderia*	NA	0.818	0.001
ASV_00338	*(p) Proteobacteria; (g) Burkholderia-Caballeronia-Paraburkholderia*	NA	0.886	0.001
ASV_00006	*(p) Proteobacteria; (g) Pelomonas*	AZ-D4	0.808	0.036
ASV_00001	*(p) Proteobacteria; (g) Ralstonia*	AZ	0.822	0.002
ASV_00106	*(p) Proteobacteria; (g) Reyranella*	NA	0.919	0.001
ASV_00200	*(p) Proteobacteria; (g) Reyranella*	NA	0.913	0.001
ASV_00176	*(p) Proteobacteria; (g) Roseiarcus*	NA	0.863	0.001
ASV_00150	*(p) Proteobacteria; (g) Sphingomonas*	AZ	0.827	0.006

ASV_00014	*(p) Proteobacteria; (o) Elsterales*	NA	0.813	0.001
ASV_00034	*(p) Proteobacteria; (o) Elsterales*	NA	0.919	0.001
ASV_00076	*(p) Proteobacteria; (o) Elsterales*	NA	0.939	0.001
ASV_00203	*(p) Proteobacteria; (o) RCP2-54*	NA	0.899	0.001
ASV_00044	*(p) Proteobacteria; (o) WD260*	NA	0.923	0.001
ASV_00047	*(p) Proteobacteria; (o) WD260*	NA	0.908	0.001
ASV_00307	*(p) Proteobacteria; (o) WD260*	NA	0.912	0.001
ASV_00102	*(p) Proteobacteria; (f) Xanthobacteraceae*	LC	0.904	0.001
ASV_00219	*(p) Verrucomicrobia; (g) Candidatus* Udaeobacter	LC	0.839	0.001
ASV_00224	*(p) Verrucomicrobia; (g) Candidatus* Udaeobacter	LC	0.941	0.001
ASV_00024	*(p) Verrucomicrobia; (g) Candidatus Xiphinematobacter*	NA	0.908	0.001
ASV_00081	*(p) Verrucomicrobia; (g) Candidatus Xiphinematobacter*	NA	0.940	0.001
ASV_00132	*(p) Verrucomicrobia; (g) Candidatus Xiphinematobacter*	NA	0.867	0.001
ASV_00137	*(p) Verrucomicrobia; (g) Candidatus Xiphinematobacter*	NA	0.986	0.001
